# Disrupting CSPG‐Driven Microglia–Astrocyte Crosstalk Enables Scar‐Free Repair in Spinal Cord Injury

**DOI:** 10.1002/advs.202516269

**Published:** 2025-11-12

**Authors:** Yufei Zheng, Zhaowei Zhang, Zezhou Fu, Qingqing Wang, Siwen Zhang, Tingyu Zhang, Meifei Zhu, Shunwu Fan, Youqing Shen, Jiajia Xiang, Xin Liu

**Affiliations:** ^1^ Department of Orthopaedic Surgery Sir Run Run Shaw Hospital Zhejiang University School of Medicine Hangzhou Zhejiang 310016 China; ^2^ Key Laboratory of Mechanism Research and Precision Repair of Orthopaedic Trauma and Aging Diseases of Zhejiang Province Hangzhou Zhejiang 310016 China; ^3^ Zhejiang Key Laboratory of Smart Biomaterials and Center for Bionanoengineering College of Chemical and Biological Engineering Zhejiang University Hangzhou Zhejiang 310027 China; ^4^ College of Animal Sciences Zhejiang University Hangzhou 310058 China

**Keywords:** chondroitin sulphate proteoglycan degradation, microglia–astrocyte crosstalk, scar‐free repair, spinal cord injury, targeted gene delivery, tissue regeneration

## Abstract

Glial scar formation represents a significant obstacle to neural regeneration following spinal cord injury (SCI), evolving from a protective glial response in the acute phase to a fibrotic and inhibitory barrier in the chronic stage. In this study, chondroitin sulphate proteoglycans (CSPGs) are identified as key regulators of scar maturation via a pathogenic microglia–astrocyte axis. CSPGs promote the transition of reactive astrocytes (RAs) into scar‐forming astrocytes (SAs) by inducing a pro‐inflammatory microglial phenotype. Mechanistically, CSPGs suppress cytochrome P450 (CYP450) enzyme activity in microglia, disrupting metabolic homeostasis and perpetuating inflammatory responses. Targeted degradation of CSPGs reprogrammes microglia toward an anti‐inflammatory state, thereby attenuating SA differentiation and fibrotic matrix deposition. To enable spatiotemporally precise intervention, a reactive oxygen species‐responsive, connective tissue growth factor‐binding fusogenic lipopolyplex for RA‐targeted delivery of the chondroitinase ABC (ChABC) gene is designed. This platform selectively degrades CSPGs at the lesion border, interrupts the maladaptive glial feedback loop, and facilitates scar‐free repair after SCI. These findings reveal a metabolic mechanism underlying glial scarring and propose a precision nanotherapeutic strategy to modulate the SCI microenvironment, thereby enhancing neuronal regeneration and functional recovery.

## Graphical abstract

This study identifies CSPGs as key drivers of glial scar maturation after spinal cord injury by reprogramming microglial metabolism and inducing astrocyte fibrosis. To address this, a reactive oxygen species‐responsive, reactive astrocyte‐targeted ChABC gene delivery system is designed to locally degrade CSPGs, precisely disrupt maladaptive glial crosstalk, and promote scar‐free tissue repair and functional recovery.

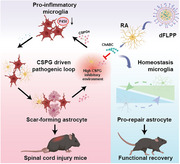

## 1 Introduction

Spinal cord injury (SCI) is a catastrophic neurological condition that often leads to permanent motor and autonomic dysfunction, with limited regenerative therapies available.^[^ ^1^ ^]^ In response to trauma, the central nervous system (CNS) mounts a complex reactive cascade involving multiple cell types, including astrocytes, microglia, oligodendrocyte precursor cells, and fibroblasts, which collectively produce a dense extracellular matrix (ECM)‐rich glial scar. Although this scar initially serves a protective function by containing inflammation and stabilizing damaged tissue, its chronic persistence presents a formidable impediment to axonal regeneration.^[^ ^2^ ^]^ Among these cellular components, astrocytes exhibit remarkable phenotypic plasticity. Upon injury, they rapidly transition into a reactive state that supports early neuroprotection. These reactive astrocytes (RAs) later differentiate into scar‐forming astrocytes (SAs), which secrete inhibitory ECM molecules and promote fibrotic scar formation.^[^ ^3^ ^]^ This phenotypic transition is thought to be modulated by signals from the local microenvironment, particularly those originating from activated microglia, which have been shown to direct astrocytic fate toward either neurotoxic or reparative phenotypes in various neurological disorders.^[^ ^4^, ^5^ ^]^ Yet, the molecular mechanisms underpinning potential microglia–astrocyte interactions after SCI and their contribution to scar persistence remain largely unclear.

Efforts to mitigate glial scarring have focused on degrading inhibitory ECM components, such as chondroitin sulphate proteoglycans (CSPGs), modulating astrocyte function through genetic or pharmacological approaches, and targeting upstream inflammatory signaling pathways.^[^ ^6^, ^7^, ^8^, ^9^, ^10^ ^]^ CSPGs, a major ECM component enriched at the lesion site, have long been implicated in restricting axonal growth.^[^ ^11^ ^]^ Once considered passive structural barriers, CSPGs are now increasingly recognized as active regulators of the post‐injury microenvironment. In addition to inhibiting neurite outgrowth, they actively influence glial cell behavior and immune responses.^[^ ^12^, ^13^ ^]^ Notably, CSPGs promote the pro‐inflammatory activation of macrophages and microglia through engagement with Toll‐like receptors (TLRs) and other pattern recognition pathways.^[^ ^12^, ^14^, ^15^ ^]^ Emerging evidence further indicates that these extracellular signals may modulate glial function through metabolic reprogramming.^[^ ^16^, ^17^, ^18^ ^]^ Within this framework, the cytochrome P450 (CYP450) enzyme family, previously appreciated for their detoxification functions in peripheral tissues,^[^ ^19^ ^]^ is gaining recognition for its role in maintaining CNS homeostasis.^[^ ^20^, ^21^, ^22^ ^]^ These enzymes govern the metabolism of lipid mediators and neurosteroids,^[^ ^23^ ^]^ thereby shaping inflammatory responses and glial identity.^[^ ^24^, ^25^ ^]^ In particular, downregulation of the CYP4F subfamily has been linked to exaggerated microglial activation and delayed resolution of neuroinflammation.^[^ ^26^ ^]^ These findings raise the possibility that CSPGs may drive scar persistence by metabolically reprogramming microglia to sustain a pro‐inflammatory state, which in turn promotes chronic astrocytic fibrosis.

Here, we uncover a pathological feed‐forward loop in which CSPGs, deposited by early RAs, suppress microglial CYP450 activity and stabilize a pro‐inflammatory microglial phenotype. These activated microglia secrete inflammatory cytokines such as tumor necrosis factor alpha (TNF‐α) and interleukin‐1 beta (IL‐1β), which subsequently induce SA differentiation and further enhance CSPG production, thereby perpetuating the cycle of chronic inflammation and fibrotic scar formation. This mechanism underscores the therapeutic potential of targeting CSPGs through enzymatic degradation, inhibition of biosynthesis, or blockade of receptor interactions (e.g., receptor‐type protein tyrosine phosphatase sigma (PTPσ), leukocyte antigen–related receptor‐type protein tyrosine phosphatase (LAR)),^[^ ^15^, ^27^, ^28^ ^]^ as a promising strategy to disrupt this self‐sustaining inflammatory cascade. Among these interventions, chondroitinase ABC (ChABC), a bacterial enzyme capable of cleaving the glycosaminoglycan chains of CSPGs, has shown significant efficacy in promoting axonal regeneration and functional recovery.^[^ ^27^, ^29^ ^]^ However, current delivery methods lack precise spatiotemporal control, risking off‐target ECM degradation and disruption of the physiological roles of CSPGs in CNS development, tissue maintenance, and aging.^[^ ^30^, ^31^ ^]^

**Figure 1:**
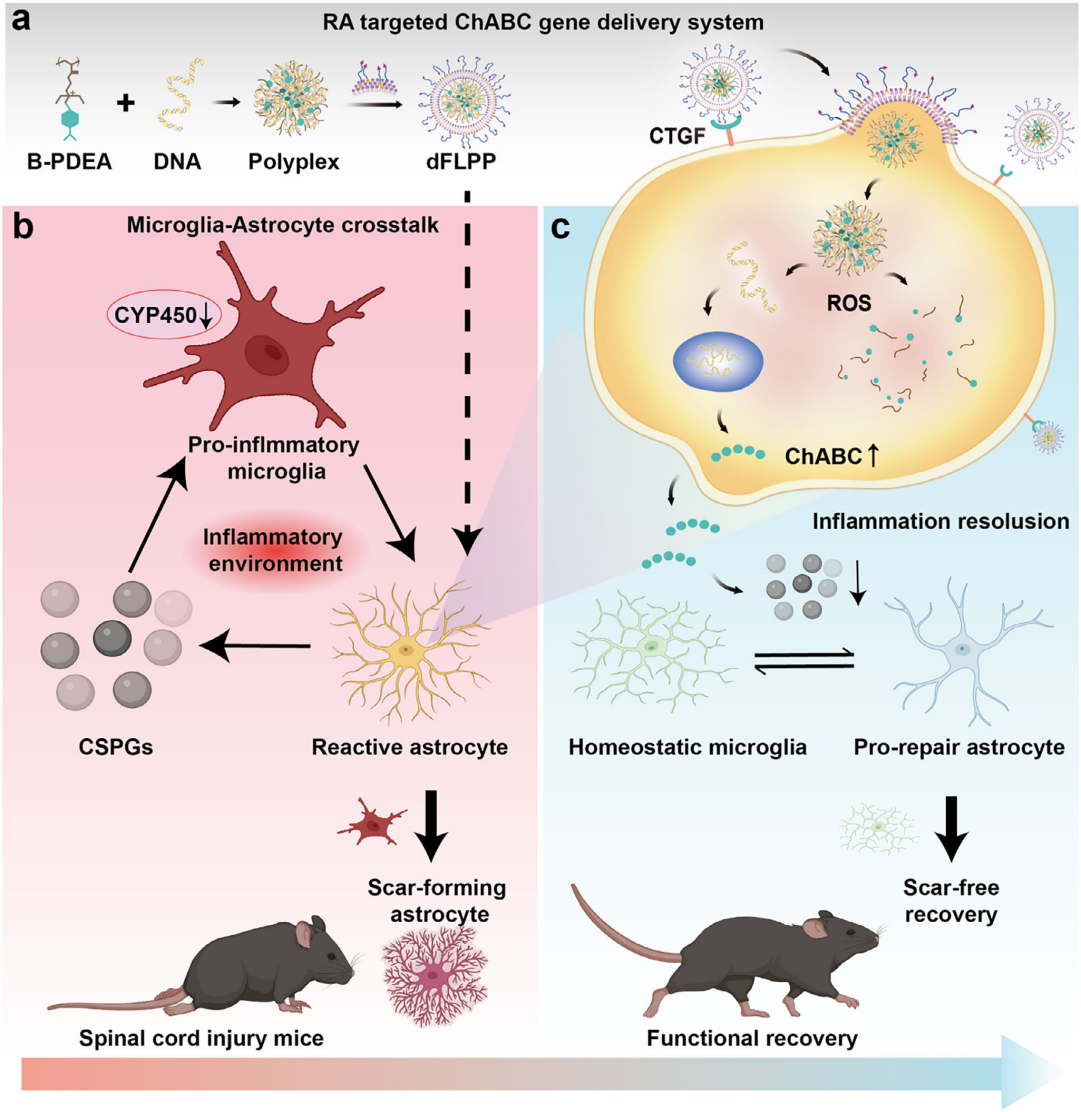
Schematic illustration of the RA‐targeted ChABC gene delivery system for scar‐free spinal cord repair. a) The dFLPP nanoplatform integrates an ROS‐responsive polymer (B‐PDEA) to condense ChABC‐encoding plasmid DNA into nanocomplexes, which are then encapsulated within fusogenic liposomes decorated with the CTGF‐binding peptide DAG. b) In the pathological feed‐forward mechanism following SCI, CSPGs inhibit CYP450 enzyme activity in microglia, leading to a pro‐inflammatory phenotype that promotes the RA‐to‐SA transition and enhances CSPG deposition, thereby perpetuating chronic glial scarring. c) The dFLPP system enables targeted delivery to RAs expressing high levels of CTGF at the lesion border. Upon RA‐specific internalization and ROS‐triggered dissociation, dFLPP promotes efficient local ChABC expression, thereby facilitating CSPG degradation, which disrupts the microglia‐astrocyte feedback loop and prevents SA differentiation. Consequently, dFLPP facilitates scar‐free tissue regeneration and supports complete functional recovery in SCI mice. Image created with BioRender.com, with permission.

To overcome these limitations, we developed a precision gene delivery platform, DAG‐decorated fusogenic lipopolyplexes (dFLPP), that enables localized, sustained, and RA‐specific expression of ChABC. The dFLPP system comprises two synergistic modules: i) a reactive oxygen species (ROS)‐responsive cationic polymer, poly [(2‐acryloyl)ethyl(p‑boronic acid benzyl)diethylammonium bromide] (B‐PDEA),^[^ ^32^, ^33^ ^]^ which condenses ChABC‐encoding plasmid DNA (pChABC) and facilitates its release under oxidative stress of SCI; and ii) a fusogenic lipid envelope functionalized with the DAG peptide (CDAGRKQKC), which selectively binds to connective tissue growth factor (CTGF), a matricellular protein highly expressed by RAs at the lesion border.^[^ ^34^ ^]^ By directing ChABC expression specifically to early CSPG‐producing RAs, dFLPP‐mediated therapy effectively disrupts the maladaptive microglia‐astrocyte feedback loop. Targeted CSPG degradation restores CYP450 enzyme activity in microglia, shifts them toward an anti‐inflammatory and reparative phenotype, and suppresses further SA differentiation and ECM deposition. As a result, this intervention preserves spinal parenchymal integrity, enhances remyelination and serotonergic circuit reactivation, and substantially improves locomotor and autonomic function in a murine model of severe contusive SCI (**Figure** **1**). Collectively, our findings identify CSPGs as active metabolic suppressors that drive glial scarring and present dFLPP as a rationally designed nanotherapeutic capable of precisely editing the post‐injury microenvironment to achieve scar‐free spinal cord regeneration.

## 2 Results

### 2.1 Early RAs Deposit CSPGs That Activate Microglia and Precede SA Emergence

**Figure 2:**
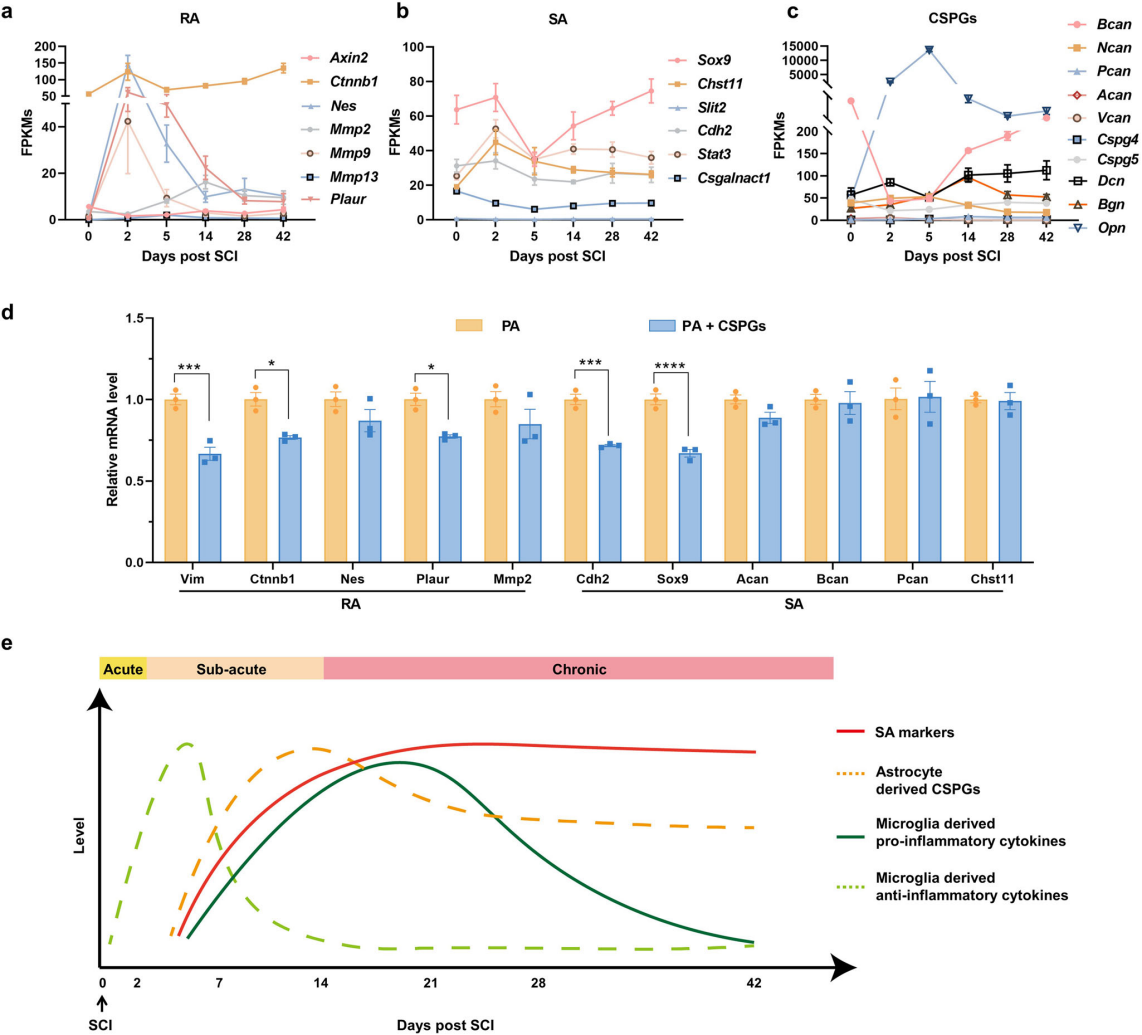
Temporal evolution of astrocyte phenotypes and CSPG expression following SCI. a–c) Expression dynamics of markers associated with RA (a), SA (b), and CSPGs (c) in Aldh1l1^+^ astrocytes across 0 to 42 dpi, based on RiboTag bulk RNA sequencing analysis. Data are presented as fragments per kilobase of transcript per million mapped reads (FPKMs); *n* = 4 mice per time point. d) qRT‐PCR analysis of RA and SA marker gene expression in PAs treated with or without CSPGs for 24 h. e) Schematic illustration depicting the spatial distribution of SA marker expression in astrocytes, secretion of astrocyte‐derived CSPGs, and microglia‐derived pro‐ and anti‐inflammatory cytokines following SCI. Data are mean ± SEM. Statistical comparisons used unpaired two‐tailed *t*‐tests.*p < 0.05, **p < 0.01, ***p < 0.001, ****p < 0.0001.

Astrocyte phenotypes are known to shift in response to SCI, but the temporal dynamics underlying this transition remain incompletely defined. To characterize the evolution of astrocyte states across different stages of SCI, we re‐analyzed a longitudinal RNA‐sequencing dataset (GSE241628) profiling Aldh1l1^+^ astrocytes, a predominant cell source defining the glial border, at multiple time points from 0 to 42 days post‐injury (dpi).^[^ ^35^ ^]^ We found that markers of the early RAs, including *Nes, Plaur*, and *Mmp9*, exhibited a transient peak during the acute phase post‐injury and declined markedly during the subacute phase (**Figure** **2**a), consistent with a transient neuroprotective response.^[^ ^3^ ^]^ In contrast, transcripts associated with SAs, such as *Sox9*, *Stat3*, and *Csgalnact1*,^[^ ^3^, ^36^ ^]^ exhibited delayed elevation (Figure 2b). Notably, a subset of SA markers, such as *Sox9* and *Stat3*, also showed a brief surge at 2 dpi, likely reflecting an acute stress response before stabilizing at elevated levels during the subacute and chronic phases, when the SA phenotype is established. These transcriptional trajectories delineate a temporal transition of astrocyte identity from reparative RA to fibrotic SA.^[^ ^37^ ^]^

Previous studies have demonstrated that RAs synthesize CSPGs in response to inflammatory signals following CNS injury.^[^ ^38^, ^39^, ^40^ ^]^ To connect astrocyte activation to CSPG deposition, we then profiled CSPG protein transcripts in Aldh1l1^+^ astrocytes across time. *Opn* (osteopontin) exhibited an acute upregulation immediately after SCI, declined in the subacute phase, and sustained high expression in the chronic phase. *Dcn* (decorin) increased gradually across the entire period. Interestingly, *Bcan* (brevican) transcript levels were acutely downregulated post‐injury, followed by a marked rebound starting at 5 dpi (Figure 2c). Although the mechanism underlying the initial decline remains unclear, the subsequent upregulation of brevican is consistent with its pathological function in promoting ECM condensation and inhibiting axonal regeneration in the chronic phase. Other CSPG subtypes exhibited either sustained high expression or varied only modestly. Overall, CSPG expression exhibited a general upward trend, consistent with their diverse functional roles after CNS injury.^[^ ^27^, ^41^ ^]^

Guided by this temporal model, we next examined whether CSPGs would promote astrocyte transition toward an SA state. Surprisingly, CSPGs alone failed to induce astrocytic differentiation into the fibrotic phenotype. When primary astrocytes (PAs) were exposed to CSPGs in vitro, transcripts associated with both the early reactive phenotype (*Vim*, *Ctnnb1*, *Nes*, *Plaur*, and *Mmp2*) and scar‐forming phenotype (*Cdh2*, *Sox9*, *Acan*, *Bcan*, *Pcan*, and *Chst11*) were either unchanged or downregulated (Figure 2d). These findings suggest that, beyond CSPGs, intermediary signaling intermediates or inflammatory mediators may be required to drive astrocyte differentiation into the SA phenotype.

To identify upstream regulators of astrocyte fate transitions, we analyzed the temporal activation of biological processes in Aldh1l1^+^ astrocytes using Gene Ontology (GO) enrichment. At 2 dpi, biosynthetic and metabolic processes dominated the transcriptional landscape, reflecting a broad stress response. Between 5 and 14 dpi, coinciding with the upregulation of *Sox9*, *Csgalnact1*, and other SA markers, we observed a clear shift toward immune‐ and cytokine‐related pathways (Figure S1, Supporting Information), indicating that astrocytes became increasingly responsive to inflammatory cues. This timing aligns with established microglial dynamics after CNS injury. Microglia and macrophages initially mount a damage‐limiting program with anti‐inflammatory and cytoprotective functions; however, ≈7 dpi, they transition toward a pro‐inflammatory state with increased secretion of TNF‐α, IL‐1β, and related mediators,^[^ ^42^, ^43^ ^]^ reaching their peak ≈2–4 weeks post‐injury.^[^ ^44^, ^45^ ^]^ Given that microglial activation is essential for mature scar formation across various CNS‐injury models,^[^ ^46^, ^47^, ^48^ ^]^ these temporally coordinated events imply that early CSPG deposition by RAs may serve as an immunomodulatory signal, converting transient microglial activation into the sustained inflammatory environment necessary for SA differentiation (Figure 2e).

### 2.2 CSPGs Reprogram Microglial Metabolism and Launch a Feed‐Forward Loop Driving SA Differentiation

**Figure 3:**
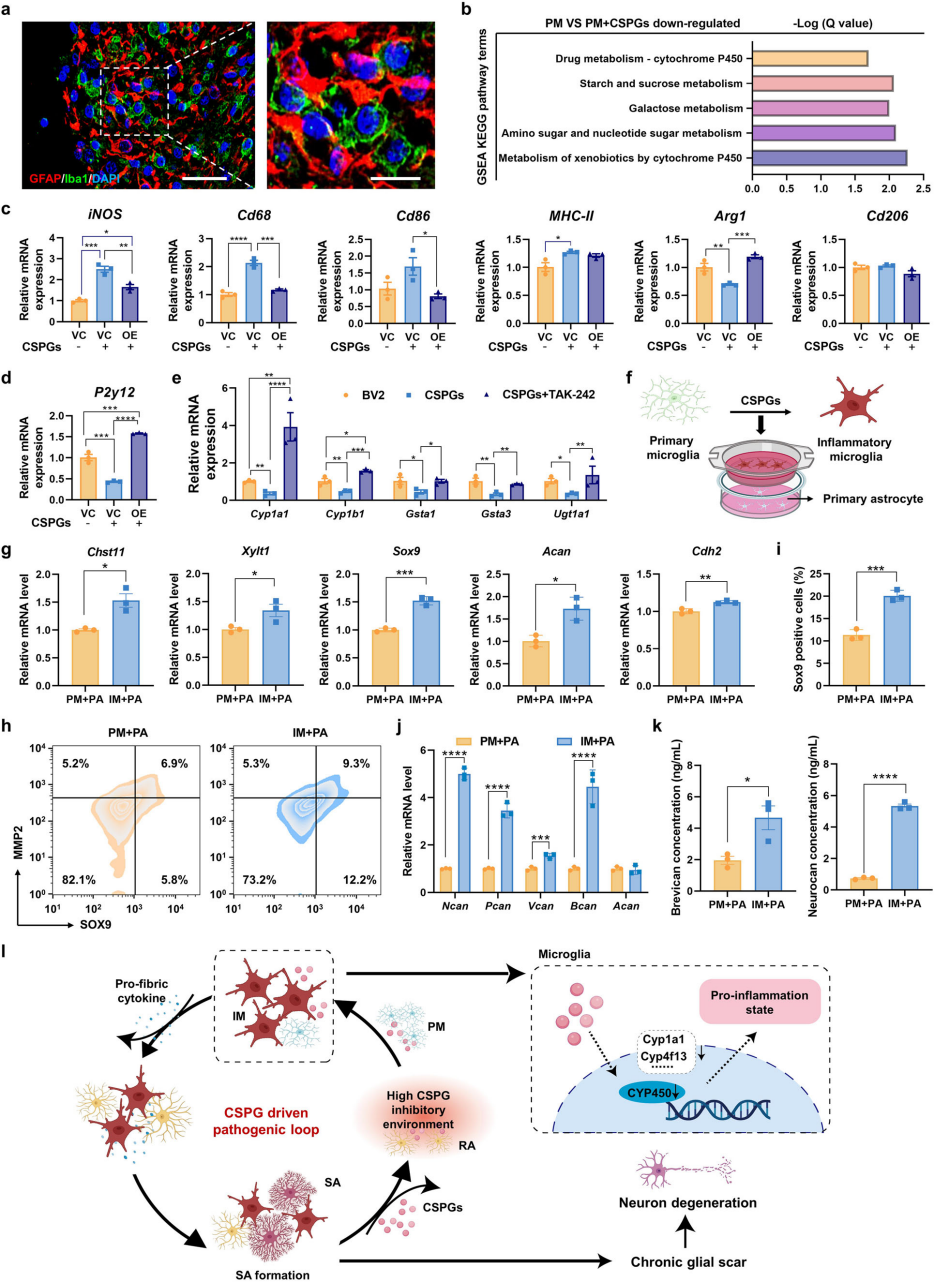
CSPGs promote SA formation by modulating microglial inflammatory responses. a) Immunofluorescence staining of spinal cord sections at the lesion border at 7 dpi showing astrocytes (GFAP^+^, green) and microglia (Iba1^+^, red). Nuclei stained with DAPI are shown in blue. Scale bars: 50 µm (left), 10 µm (right). b) GSEA showing significant downregulation of CYP450‐associated pathways in PM following 48 h of CSPG stimulation. PMs were treated with or without CSPGs (3.34 µg mL^−1^) for 72 h (*n* = 3). c,d) qRT‐PCR analysis of inflammatory markers in BV2 microglia transfected with a *Cyp1a1* overexpression plasmid (1 µg mL^−1^) and subsequently stimulated with CSPGs (3.34 µg mL^−1^, 48 h) (*n* = 3). e) qRT‐PCR analysis of CYP450‐related genes in BV2 cells treated with CSPGs (3.34 µg mL^−1^, 24 h) with or without the TLR4 inhibitor TAK‐242 (50 nm) (*n* = 3). f) Schematic of the transwell co‐culture system. PM pre‐treated with CSPGs (3.34 µg mL^−1^, 24 h) to generate IMs were placed in the upper chamber and co‐cultured with PAs in the lower chamber for 48 h. g) qRT‐PCR analysis of SA marker genes in astrocytes after 48 h of co‐culture with IMs (*n* = 3). h,i) Flow cytometric analysis of astrocyte phenotypes showing proportions of SOX9^+^ (SA‐like) and MMP2^+^ (RA‐like) cells (h) and quantification of the SOX9^+^ population (i) (*n* = 3). j) qRT‐PCR analysis of CSPG core protein genes in astrocytes after 48 h co‐incubation with CSPG‐stimulated or unstimulated microglia (*n* = 3). k) ELISA quantification of secreted brevican and neurocan in conditioned media after 48 h co‐culture with IMs (*n* = 3). l) Schematic illustration of the CSPG‐driven feed‐forward loop promoting glial scar formation. Image created with BioRender.com, with permission. Data are mean ± SEM. Statistical comparisons used unpaired two‐tailed *t*‐tests (two groups) or one‐way ANOVA with Tukey's post hoc test (≥3 groups). *p < 0.05, **p < 0.01, ***p < 0.001, ****p < 0.0001.

Beyond serving as structural and biochemical barriers to axonal regeneration, CSPGs may function as paracrine modulators of glial crosstalk after injury. At 7 dpi, GFAP⁺ astrocytes and Iba1⁺ microglia interwove tightly along the lesion border (**Figure** **3**a), positioning these cells in proximity for bidirectional signaling. Thus, we hypothesized that excess CSPGs might first reprogram microglia and thereby influence astrocyte fate. To test this, we exposed primary microglia (PM) to purified CSPGs. This stimulation provoked a strong pro‐inflammatory response, characterized by the upregulation of microglial activation genes, including *Cxcl3*, *Edn1*, and *Cd69* (Figure S2a, Supporting Information).^[^ ^49^ ^]^ Gene set enrichment analysis (GSEA) revealed concurrent downregulation of multiple CYP450 modules (Figure 3b). Because the CYP450 system detoxifies oxidized lipids, steroids, and xenobiotics, its suppression implies a metabolic shift that removes an intrinsic anti‐inflammatory brake.^[^ ^50^, ^51^ ^]^ Transcriptomic profiling corroborated this shift. Key phase I detoxification enzymes (*Cyp1a1*, *Cyp2d22*) and phase II conjugation enzymes (*Ugt1a1*, *Ugt1a6*) were significantly downregulated (Figure S2b, Supporting Information), predicting impaired oxidative and glucuronidation pathways and, consequently, the accumulation of lipid peroxides and eicosanoid‐like mediators that lower the activation threshold of microglia.

CSPG‐mediated inhibition of detoxification pathways exhibits both dose‐ and time‐dependence. Increasing CSPG concentrations progressively suppressed transcripts of *Cyp1a1*, *Cyp1b1*, and *Cyp2d22*, and time‐course analyses revealed a sustained and gradually intensifying repression of these xenobiotic‐metabolizing genes (Figure S3, Supporting Information). Moreover, we performed gain‐of‐function rescue experiments in BV2 microglia. Overexpression of *Cyp1a1*, a member of the CYP450 superfamily, significantly attenuated CSPG‐induced inflammation, lowering the expression of pro‐inflammatory mediators, including *iNOS*, *Cd68*, and *Cd86*, while elevating levels of anti‐inflammatory marker *Arg1* and the homeostatic and surveillant microglia hallmark *P2y12* (Figure 3c,d; Figure S4, Supporting Information), indicating reversion toward a neuroprotective phenotype.^[^ ^52^, ^53^ ^]^ Conversely, pharmacological inhibition of CYP450 activity using bergamottin exacerbated inflammatory responses following lipopolysaccharide (LPS) challenge, upregulating *iNOS*, *CD68*, and *CD86* while downregulating *Arg1* (Figure S5, Supporting Information). These findings demonstrate that suppression of CYP450 activity is a critical mechanism underlying CSPG‐induced inflammatory activation in microglia.

We next investigated the mechanism by which CSPGs suppress the CYP450 pathway. Previous studies have demonstrated that TLR4 signaling plays a critical role in regulating CYP450 signaling,^[^ ^54^ ^]^ and its expression is upregulated following exposure to CSPGs.^[^ ^12^ ^]^ To assess whether TLR4 signaling is involved, we measured the expression of key CYP450‐related genes in microglia treated with CSPGs in the presence or absence of a pharmacological TLR4 inhibitor. Notably, co‐treatment with the TLR4 inhibitor TAK‐242 significantly reversed the suppression of the CYP450 pathway induced by CSPGs, restoring expression levels of *Cyp1a1* and *Cyp1b1*, as well as other detoxification genes, including *Gsta1*, *Gsta3*, and *Ugt1a1*, to near or above baseline levels (Figure 3e). These findings indicate that TLR4 signaling mediates the inhibitory effects of CSPGs on the CYP450 pathway.

To determine whether CSPG‐activated microglia influence astrocyte identity, we employed a transwell co‐culture system, in which PAs were exposed for 48 h to inflammatory microglia (IMs) pre‐primed with CSPGs (Figure 3f). In the absence of direct cell contact, astrocytes exhibited upregulation of multiple SA‐associated genes, including *Chst11*, *Xylt1*, *Sox9*, *Acan*, and *Cdh2* (Figure 3g), alongside a significant increase in the proportion of SOX9^+^ astrocytes (Figure 3h). Meanwhile, CSPG‐stimulated microglia expressed elevated levels of pro‐fibrotic cytokines, such as *Col1a1*, *Col2a1*, *Tnf‐α*, and *Il‐1β* (Figure S6, Supporting Information), all of which have been previously implicated in promoting SA differentiation.^[^ ^3^, ^9^, ^55^, ^56^ ^]^ These findings suggest that microglia translate extracellular CSPG signals into inflammatory cytokine responses that alter astrocyte identity and promote fibrotic reprogramming. Although modest increases in RA‐associated transcripts (*Axin*, *Mmp9*, *Ctnnb1*) were detected (Figure S7, Supporting Information), flow cytometric analysis revealed that SOX9^+^ SAs greatly outnumbered MMP2^+^ RAs (Figure 3i; Figure S8, Supporting Information), further supporting a predominant shift toward a scar‐forming phenotype under the influence of CSPG‐activated microglia.

To assess whether SAs contribute to further CSPG deposition, we quantified the expression and secretion of CSPG core proteins in the co‐culture system. Astrocytes exposed to CSPG‐primed microglia exhibited increased transcript levels of *Ncan*, *Pcan*, *Vcan*, and *Bcan* (Figure 3j), and enzyme‐linked immunosorbent assay (ELISA) revealed elevated concentrations of secreted brevican and neurocan in the culture medium (Figure 3k). These results delineate a pathogenic feed‐forward mechanism (Figure 3l): CSPGs deposited by early RAs impair microglial CYP450 activity, leading to a pro‐inflammatory secretome enriched in TNF‐α and IL‐1β. These cytokines subsequently induce neighboring astrocytes to adopt an SA phenotype, which actively synthesizes additional CSPGs, thereby perpetuating microglial activation. This escalating cycle of metabolic dysfunction, inflammatory signaling, and ECM deposition offers a mechanistic rationale for chronic scar progression after SCI. Therapeutic interventions targeting this loop by restoring microglial CYP450 function, neutralizing fibrogenic cytokines, or locally degrading CSPGs could potentially halt scar expansion and facilitate neural regeneration.

### 2.3 A ROS‐Responsive, RA‐Targeted Nanoplatform Enables Efficient ChABC Expression

Given the critical physiological role of CSPGs in maintaining tissue architecture and cell–matrix interactions, indiscriminate degradation may pose a risk to adjacent healthy tissues. Thus, a targeted strategy that restricts CSPG removal to the site of injury is essential. RAs, activated during the acute phase post‐injury, represent the primary source of early CSPG production at the lesion periphery, making them an ideal target for spatially confined therapeutic intervention. CTGF, a matricellular protein involved in cell proliferation, ECM remodeling, and scar formation, is significantly upregulated in RAs.^[^ ^34^ ^]^ Immunostaining further confirmed CTGF enrichment at the injury border (Figure S9, Supporting Information), supporting its potential as a molecular handle for RA‐specific targeting.

**Figure 4:**
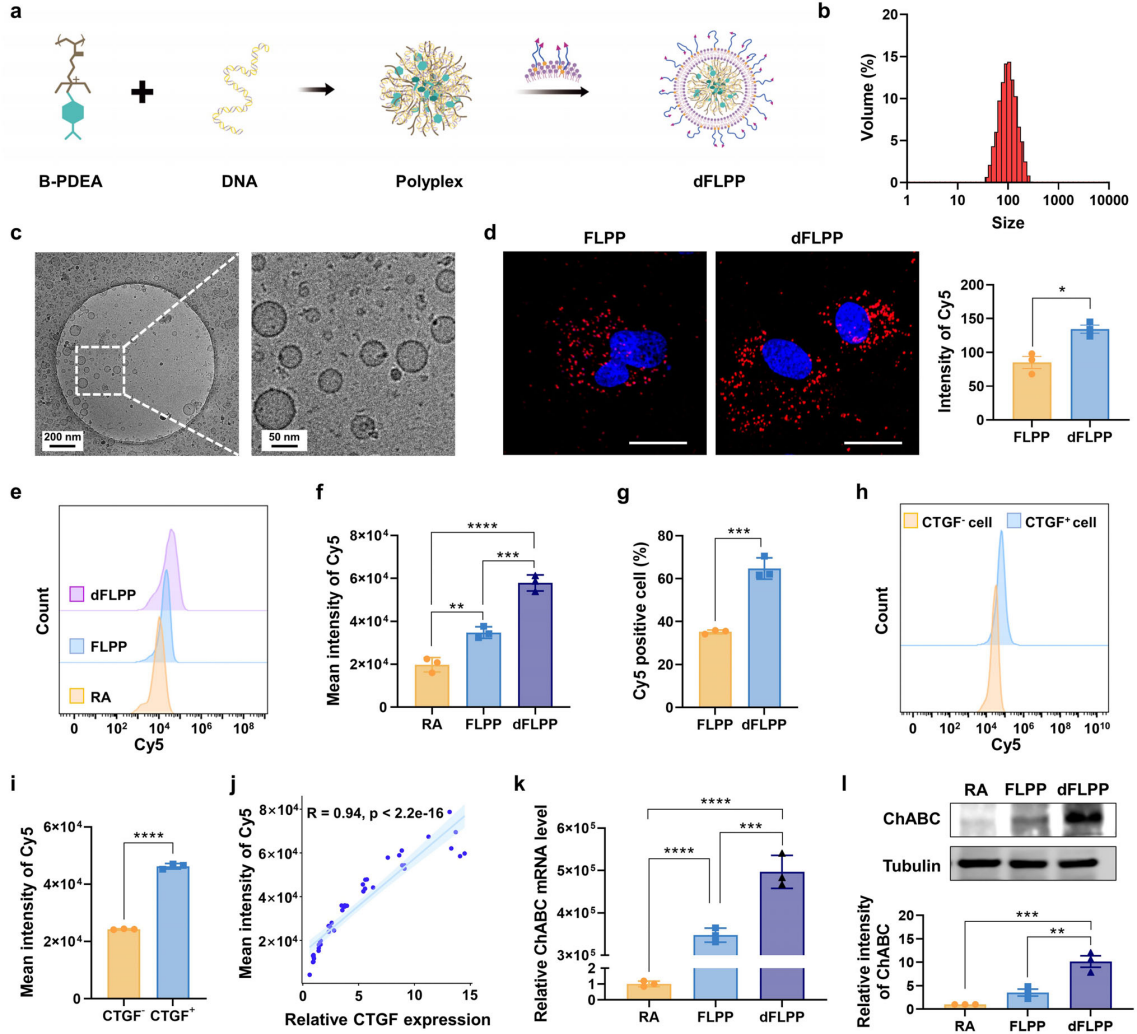
Preparation, characterization, and RA‐targeting capability of dFLPP. a) Schematic illustration of the construction of dFLPP. b) Hydrodynamic diameter of dFLPP measured by dynamic light scattering. c) Representative cryo‐electron microscopy images showing the morphology of dFLPP. Scale bars: 200 nm (left), 50 nm (right). d) Confocal microscopy images and corresponding fluorescence quantification of dFLPP and FLPP uptake by LPS‐stimulated RAs after 1 h of incubation (*n* = 3); ^Cy5^DNA, 1 µg mL^−1^. Scale bar: 20 µm. e–g) Flow cytometric analysis of cellular uptake in RAs after 1 h incubation with FLPP or dFLPP, showing representative histograms (e), intracellular fluorescence intensity (f), and percentage of Cy5‐positive cells (g) (*n* = 3). ^Cy5^DNA, 100 ng mL^−1^. h,i) Flow cytometric analysis of dFLPP uptake in a mixed astrocyte population containing PAs and RAs after 1 h co‐incubation with dFLPP; ^Cy5^DNA, 100 ng mL^−1^. j) Correlation analysis between intracellular Cy5 fluorescence intensity and CTGF expression levels in a mixed population of RAs and PAs using Pearson correlation analysis. CTGF expression was assessed by flow cytometric analysis following immunofluorescent labeling using a primary anti‐CTGF antibody and a FITC‐labeled secondary antibody (*n* = 6). k,l) ChABC protein expression (k) and mRNA levels (l) in RAs transfected with dFLPP or FLPP for 48 h (*n* = 3); pChABC dose, 2 µg mL^−1^. Data are mean ± SEM. Statistical comparisons used unpaired two‐tailed *t*‐tests (two groups) or one‐way ANOVA with Tukey's post hoc test (≥ 3 groups). *p < 0.05, **p < 0.01, ***p < 0.001, ****p < 0.0001.

Building on this foundation, we engineered a precision gene delivery platform, termed DAG‐decorated fusogenic lipopolyplex (dFLPP), to enable selective expression of ChABC, a bacterial CSPG‐degrading enzyme, specifically within RA‐enriched regions. The dFLPP platform integrates two synergistic modules: i) B‐PDEA, a ROS‐responsive cationic polymer that condenses ChABC‐encoding plasmid DNA (pChABC) into nanopolyplexes and facilitates their release in response to the oxidative stress characteristic of the SCI microenvironment; and ii) a fusogenic lipid envelope functionalized with the RA‐targeting peptide CDAGRKQKC (DAG), which selectively binds to CTGF‐expressing astrocytes (**Figure** **4**a). This dual‐functional design enables both spatial targeting and conditional activation, thereby minimizing off‐target effects and allowing precise modulation of the fibrotic niche.

Self‐assembly of B‐PDEA and pChABC formed spherical polyplexes with diameters ranging from 50 to 100 nm and zeta potentials of +11 to +15 mV at N/P ratios above 5 (Figure S10a, Supporting Information). In transfection assays using a luciferase reporter plasmid (pLuc), these polyplexes achieved maximal gene expression at an N/P of 10 in both PAs and BV2 cells, with transfection efficiency surpassing that of the benchmark polymer polyethyleneimine (PEI, 25 kDa) by two orders of magnitude (Figure S10b,c, Supporting Information). To confer in vivo stability and RA‐specific targeting, the polyplexes were further encapsulated within a fusogenic lipid envelope composed of 1,2‐dioleoyl‐sn‐glycero‐3‐phosphoethanolamine (DOPE), cholesteryl hemisuccinate (CHEMS), and DAG‐conjugated distearoyl phosphoethanolamine‐polyethylene glycol (DSPE‐PEG‐DAG). The final construct, dFLPP, displayed a hydrodynamic diameter of ≈100 nm and a surface charge reversal to −6 mV due to PEG shielding (Figure 4b), consistent with the vesicular morphology observed via cryogenic electron microscopy (cryo‐EM) (Figure 4c). Gel retardation assays showed that dFLPP stably retained plasmid DNA under physiological conditions but efficiently released it in response to H_2_O_2_ exposure, enabling ROS‐triggered gene delivery in the SCI region (Figure S11, Supporting Information).

To validate the RA‐targeting capability of dFLPP, we incorporated Cy5‐labeled plasmid (^Cy5^DNA) into dFLPP or control lipopolyplexes lacking DAG (FLPP) and applied these formulations to LPS‐stimulated RAs. Confocal microscopy revealed significantly enhanced cellular uptake of dFLPP, as evidenced by stronger intracellular Cy5 fluorescence compared to FLPP (Figure 4d; Figure S12, Supporting Information). These observations were corroborated by flow cytometry, which showed that 64.8% of RAs internalized dFLPP within 1 h, compared to 35.2% for FLPP (Figure 4e–g), thereby confirming the improved delivery efficiency conferred by DAG functionalization. To further assess the dependence on CTGF expression, dFLPP uptake was evaluated in a heterogeneous astrocyte population containing both PAs and LPS‐stimulated RAs. Flow cytometric analysis demonstrated a preferential uptake of dFLPP by CTGF^+^ subpopulations (Figure 4h,i; Figure S13, Supporting Information), with a strong linear correlation observed between CTGF expression levels and Cy5 signal intensity (Figure 4j). These findings substantiate the spatial selectivity of dFLPP for CTGF‐rich RAs, supporting its capacity for targeted delivery to SCI regions.

Mechanistically, dFLPP uptake was found to occur through a membrane fusion pathway that circumvents endosomal entrapment, thereby enhancing the nuclear delivery of plasmid DNA. At 4 h post‐incubation, ^Cy5^DNA and DiI‐labeled lipids co‐localized at the plasma membrane, suggesting initial membrane binding and fusion. By 18 h, Cy5 fluorescence had accumulated in the nucleus, whereas DiI fluorescence remained at the cell periphery, with minimal overlap with lysosomal markers (Figure S14, Supporting Information). This spatial distribution supports an endosome‐independent mechanism of cytosolic entry, which bypasses lysosomal degradation, a major barrier to effective gene transfection. These findings align with our previous characterization of FLPP‐mediated membrane fusion and further highlight the efficiency of the dFLPP platform in promoting intracellular gene delivery.^[^ ^32^ ^]^ Functionally, this fusion‐based internalization resulted in significantly higher ChABC expression in RAs treated with dFLPP compared to those treated with FLPP, as confirmed by qRT‐PCR and western blotting analysis (Figure 4k,l). Together, these results demonstrate that the dFLPP nanoplatform, which combines CTGF‐guided targeting, membrane fusion entry, and ROS‐triggered DNA release, enables efficient and selective transfection of RAs within the SCI microenvironment, thereby promising precise and localized CSPG degradation.

### 2.4 dFLPP Targets RAs In Vivo and Facilitates CSPG Degradation

**Figure 5:**
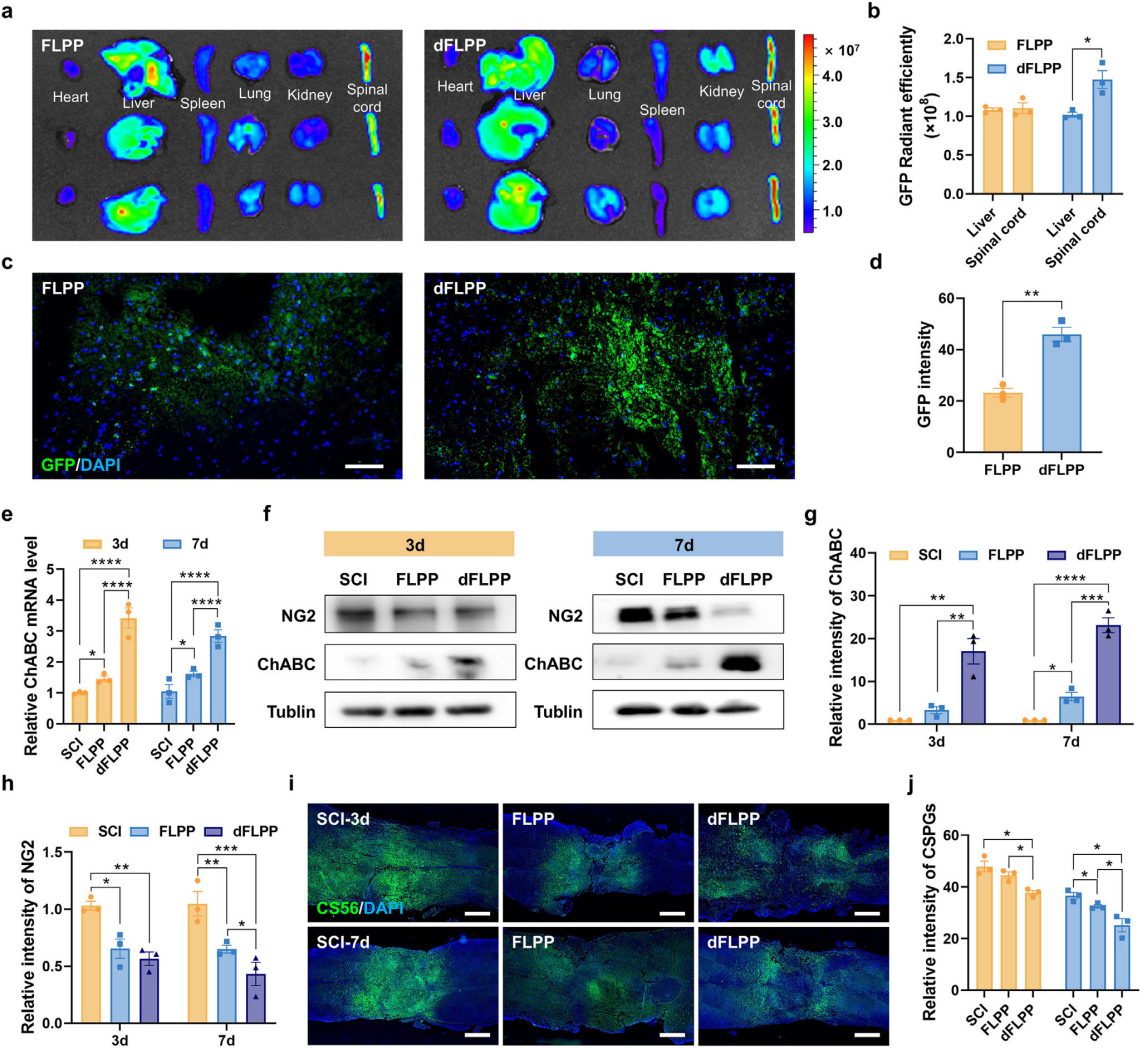
In vivo biodistribution and CSPG degradation efficiency of dFLPP. a,b) *Ex vivo* fluorescence imaging of harvested organs 48 h after intravenous (*i.v*.) injection of FLPP or dFLPP; pEGFP, 0.5 mg kg^−1^. (a), with quantification of GFP radiant efficiency in the liver and spinal cord from each group (b) (*n* = 3). c,d) GFP fluorescence imaging of lesioned spinal cord sections 48 h post‐treatment (c), with corresponding quantification of GFP intensity (d) (*n* = 3). Scale bar: 50 µm. e) ChABC mRNA expression in the lesioned spinal cord at 3 and 7 dpi across SCI, FLPP, and dFLPP groups. FLPP or dFLPP was *i.v*. administered at 1 dpi and repeated every other day for a total of one or three doses (*n* = 3); pChABC dose, 0.5 mg kg^−1^. f–h) Western blot analysis of ChABC and NG2 protein levels in the lesioned spinal cord at 3 and 7 dpi (f), with corresponding quantification (g,h) (*n* = 3). i,j) Immunofluorescence staining of CS56 in spinal cord lesions at 3 and 7 dpi (i) and corresponding fluorescence quantification (j) (*n* = 3). Scale bar: 100 µm. Data are mean ± SEM. Statistical comparisons used unpaired two‐tailed *t*‐tests (two groups) or one‐way ANOVA with Tukey's post hoc test (≥3 groups). *p < 0.05, **p < 0.01, ***p < 0.001, ****p < 0.0001.

Precise delivery of therapeutic genes to the border of fibrotic lesions is crucial for effectively degrading pathological CSPGs while safeguarding adjacent healthy tissue. To assess in vivo targeting efficiency, we intravenously (*i.v*.) administered DiI‐labeled dFLPP or control FLPP, each encapsulating a plasmid encoding enhanced green fluorescent protein (pEGFP), into SCI mice at 1 dpi. In vivo imaging revealed substantial DiI accumulation in the injured spinal cords of dFLPP‐treated mice, indicating enhanced lesion tropism mediated by DAG‐targeting of CTGF^+^ RAs (Figure S15a, Supporting Information). Quantitative analysis of *ex vivo* fluorescence at 24 h post‐injection confirmed ≈1.5‐fold higher signal intensity in the spinal cord compared to controls (Figure S15b–e, Supporting Information). Although DiI signals were also observed in the liver, likely due to normal physiological clearance, no off‐target transgene expression was detected. Notably, EGFP expression in the spinal cords of dFLPP‐treated mice was significantly increased, with fluorescence intensity ≈1.4 times greater than that in the FLPP group and 1.5 times higher than hepatic expression, thereby validating site‐specific gene transfection (**Figure** **5**a,b). Fluorescence microscopy of spinal cord sections further supported these findings, revealing strong localized EGFP expression specifically in the dFLPP group (Figure 5c,d). Collectively, these results demonstrate that dFLPP facilitates efficient and lesion‐restricted gene delivery through the synergistic combination of RA‐targeting and ROS‐responsive payload release.

Having established lesion‐restricted delivery, we next asked whether this selectivity reflected preferential uptake by CTGF^+^ RAs at the lesion border. Previous studies have shown that astrocytes and endothelial cells are the primary CTGF‐expressing populations in the injured spinal cord.^[^ ^43^, ^57^, ^58^ ^]^ To determine cell‐type specificity in vivo, we administered Rhodamine B (RhoB)‐labeled dFLPP at 1 dpi and dissociated spinal cords 24 h later for flow cytometric analysis. GFAP^+^ astrocytes exhibited significantly higher Rhodamine B fluorescence intensity and a greater positivity rate compared to CD31^+^ endothelial cells (Figure S16a, Supporting Information), indicating preferential astrocytic targeting. Two factors likely underpin this selectivity: i) astrocytes constitute a larger fraction of CTGF‐expressing cells than endothelial cells in the injured spinal cord, as indicated by analyses from the Tabulae Paralytica dataset^[^ ^58^ ^]^ and further corroborated by our GFAP/CD31 immunostaining results (Figure S16b, Supporting Information); and ii) CTGF levels are significantly higher in astrocytes than in endothelial cells, consistent with our in vitro finding that dFLPP uptake is positively correlated with CTGF expression levels (Figure S16c, Supporting Information).

Subsequently, we evaluated whether dFLPP‐mediated gene delivery translated into functional ChABC production and effective CSPG degradation in vivo. qRT‐PCR analysis demonstrated a significant increase in ChABC mRNA levels in the dFLPP‐treated spinal cords, approximately twofold higher than those in the FLPP control group at both 3 and 7 dpi (Figure 5e). This upregulation at the transcriptional level was reflected at the protein level, as evidenced by Western blotting showing approximately fivefold higher ChABC expression in the dFLPP group compared to the FLPP‐treated mice (Figure 5f,g). Crucially, this enzymatic activity resulted in the selective degradation of CSPGs, which are involved in scar maintenance. NG2 (*Cspg4*), a transmembrane CSPG known to maintain scar rigidity and inhibit axonal regeneration during the acute and subacute phases, exhibited significantly reduced expression following dFLPP treatment at both 3 and 7 dpi (Figure 5f,h). This enhanced CSPG degradation was further corroborated by a decrease in immunoreactivity for CS56, a pan‐CSPG epitope that recognizes glycosaminoglycan chains (Figure 5i,j). Additionally, transcript levels of brevican (*Bcan*) and neurocan (*Ncan*), both of which are typically upregulated in response to inflammatory mediators, were markedly downregulated in dFLPP‐treated spinal cords by 7 dpi (Figure S17, Supporting Information). These observations suggest that dFLPP‐mediated ChABC overexpression not only facilitates the degradation of extracellular CSPGs but also disrupts astrocytic pathways responsible for CSPG synthesis under chronic inflammatory conditions. Together, these results demonstrate that dFLPP enables precise targeting of the injured spinal cord, induces robust and localized ChABC expression, and remodels the ECM by effectively eliminating both matrix‐bound and astrocyte‐associated CSPG pools.

### 2.5 dFLPP Reprograms Microglial Phenotype and Metabolism to Support Inflammation Resolution

**Figure 6:**
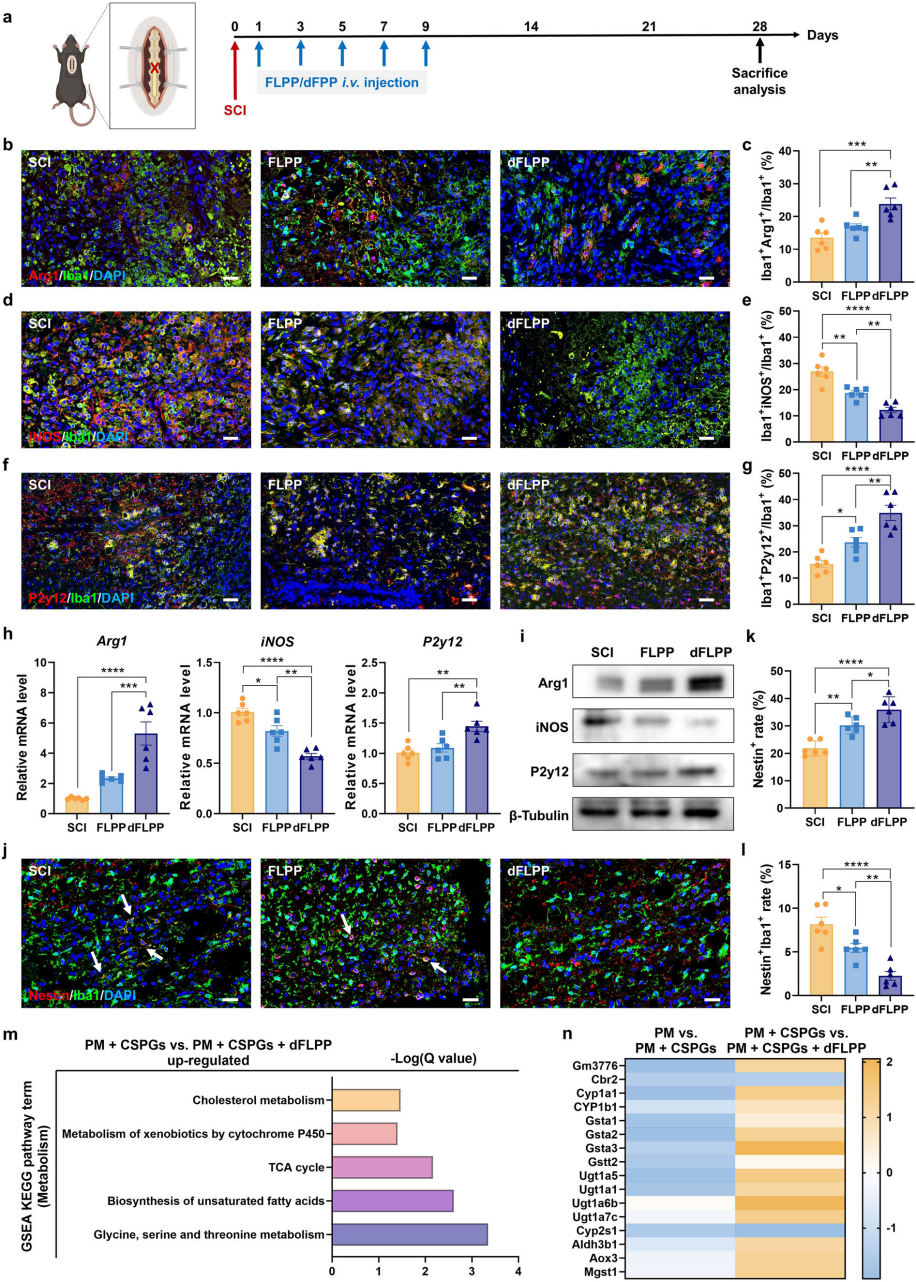
RA‐targeted CSPG degradation by dFLPP modulates microglial activity after SCI. a) Schematic illustration of the treatment regimen. FLPP or dFLPP was *i.v*. administered at 1 dpi in SCI mice every other day for a total of five doses; pChABC dose, 0.5 mg kg^−1^. b–e) Immunofluorescence staining of pro‐inflammatory (iNOS^+^; red) (b) and anti‐inflammatory (Arg1^+^; red) (d) microglia in spinal cord lesions at 7 dpi, co‐stained with Iba1 (green) and nuclei (DAPI; blue), and corresponding quantification of iNOS^+^ (c) and Arg1^+^ (e) microglial populations (*n* = 6). Scale bar: 20 µm. f,g) Immunofluorescence staining (f) and quantification (g) of homeostatic microglia (P2y12^+^; red) at 28 dpi, co‐stained with Iba1 (green) and DAPI (blue) (*n* = 6). Scale bar: 20 µm. h) Relative mRNA expression levels of *iNOS*, *Arg1*, and *P2y12* in spinal cords across treatment groups at 7 dpi (*n* = 6). i) Western blot analysis of iNOS, Arg1, and P2y12 protein levels in spinal cords across treatment groups at 7 dpi. j–l) Immunofluorescence images (j) and quantification of Nestin^+^ cells (k) and Nestin^+^Iba1^+^ microglia (l) in spinal cord lesions at 7 dpi. Nestin (red), Iba1 (green), and nuclei (blue) (*n* = 6). Scale bar: 20 µm. m) GSEA showing significantly upregulated gene pathways in CSPG‐stimulated PM treated with or without dFLPP (*n* = 3). PMs were treated with CSPGs (3.34 µg mL^−1^) or co‐treated with CSPGs and dFLPP (2 µg mL^−1^ pChABC) for 72 h in vitro. n) Heatmap illustrating relative expression levels of CYP450‐associated metabolic genes following the indicated treatments (*n* = 3). PMs were either left untreated, treated with CSPGs (3.34 µg mL^−1^), or co‐treated with CSPGs and dFLPP (2 µg mL^−1^ pChABC) for 72 h *in vitr*o. Data are mean ± SEM. Statistical comparisons used one‐way ANOVA with Tukey's post hoc test (≥3 groups). *p < 0.05, **p < 0.01, ***p < 0.001, ****p < 0.0001.

Microglia are highly plastic immune sentinels whose phenotype is shaped by local cues after SCI. Because CSPGs sustain chronic microglial activation and dFLPP depletes CSPGs in vivo, we asked whether ECM remodeling would reprogram microglia toward an inflammation‐resolving state (**Figure** **6**a). At 7 dpi, immunofluorescence analysis revealed a substantial phenotypic shift in microglia following dFLPP treatment. Specifically, the proportion of Iba1^+^Arg1^+^ microglia, indicative of anti‐inflammatory and tissue‐repairing phenotypes, was significantly increased. In parallel, the population of Iba1^+^iNOS^+^ microglia, associated with pro‐inflammatory activation, was markedly reduced compared to both FLPP‐treated and untreated controls (Figure 6b–e). Notably, this immunomodulatory effect persisted into the chronic phase. By 28 dpi, the number of P2y12^+^ microglia, indicative of homeostatic surveillance, was significantly restored in the dFLPP group (Figure 6f,g), suggesting a durable transition to a neuroprotective state rather than transient immunosuppression. This phenotypic transformation was further supported by transcriptional and protein‐level analyses of Arg1, iNOS, and P2y12 (Figure 6h,i; Figure S18, Supporting Information). To contextualize these shifts, we evaluated a panel of gene expression across three functional dimensions in vitro. CSPG stimulation downregulated homeostatic markers (*P2y12*, *Tmem119*, *Cx3cr1*, and *Sall1*) and cytoprotective genes (*Nrf2*, *Hmox1*, and *Pink1*), while upregulating genes associated with oxidative stress and glycolytic activation (*Hif1a*, *Hk2*, and *Glut1*). Notably, dFLPP treatment substantially reversed these alterations (Figure S19, Supporting Information). These findings suggest that CSPG clearance by dFLPP rebalanced the lesion microenvironment, redirecting microglia from inflammatory activation toward reparative function.

Beyond its direct effects on microglia, dFLPP treatment also reshaped the broader cellular milieu. We observed an increase in the number of Nestin^+^ cells within the lesion area, suggesting either recruitment or proliferation of regenerative neural progenitors.^[^ ^59^ ^]^ Concomitantly, the proportion of Nestin^+^Iba1^+^ microglia, a cell population previously linked to the heightened neuroinflammatory activity^[^ ^60^ ^]^ was significantly reduced in dFLPP‐treated spinal cords (Figure 6j–l). These data indicate that dFLPP not only dismantles structural barriers to regeneration but also recalibrates cellular phenotypes to favor immune homeostasis and tissue repair.

To determine whether these effects directly result from ECM normalization acting on microglia, we treated CSPG‐exposed PM with dFLPP in vitro. Transcriptomic profiling revealed extensive changes in gene expression following dFLPP‐mediated CSPG degradation, including the upregulation of anti‐inflammatory markers (Figure S20, Supporting Information). These findings support a direct and cell‐intrinsic response to ECM normalization, independent of secondary signaling from other cell types. Further mechanistic insight was gained through GSEA, which revealed restoration of CYP450 metabolic activity following dFLPP treatment (Figure 6m). Specifically, dFLPP reactivated the expression of phase I detoxification enzymes (e.g., *Cyp1a1*), phase II conjugation enzymes (e.g., *Ugt1a5*), and multiple glutathione S‐transferase family members (e.g., *Gsta1‐3*) (Figure 6n). These enzymes are crucial for maintaining redox homeostasis, metabolizing reactive lipid intermediates, and resolving inflammation.^[^ ^19^, ^61^, ^62^, ^63^ ^]^ The restoration of CYP450 activity implies that CSPGs impose a metabolically suppressive state that sustains microglial activation. By alleviating this constraint, dFLPP enables microglia to revert to a homeostatic and reparative phenotype, thereby fostering a regenerative environment in the injured spinal cord.

### 2.6 dFLPP Modulates Astrocyte‐Microglia Interactions to Reduce SA Formation and Scar Deposition

**Figure 7:**
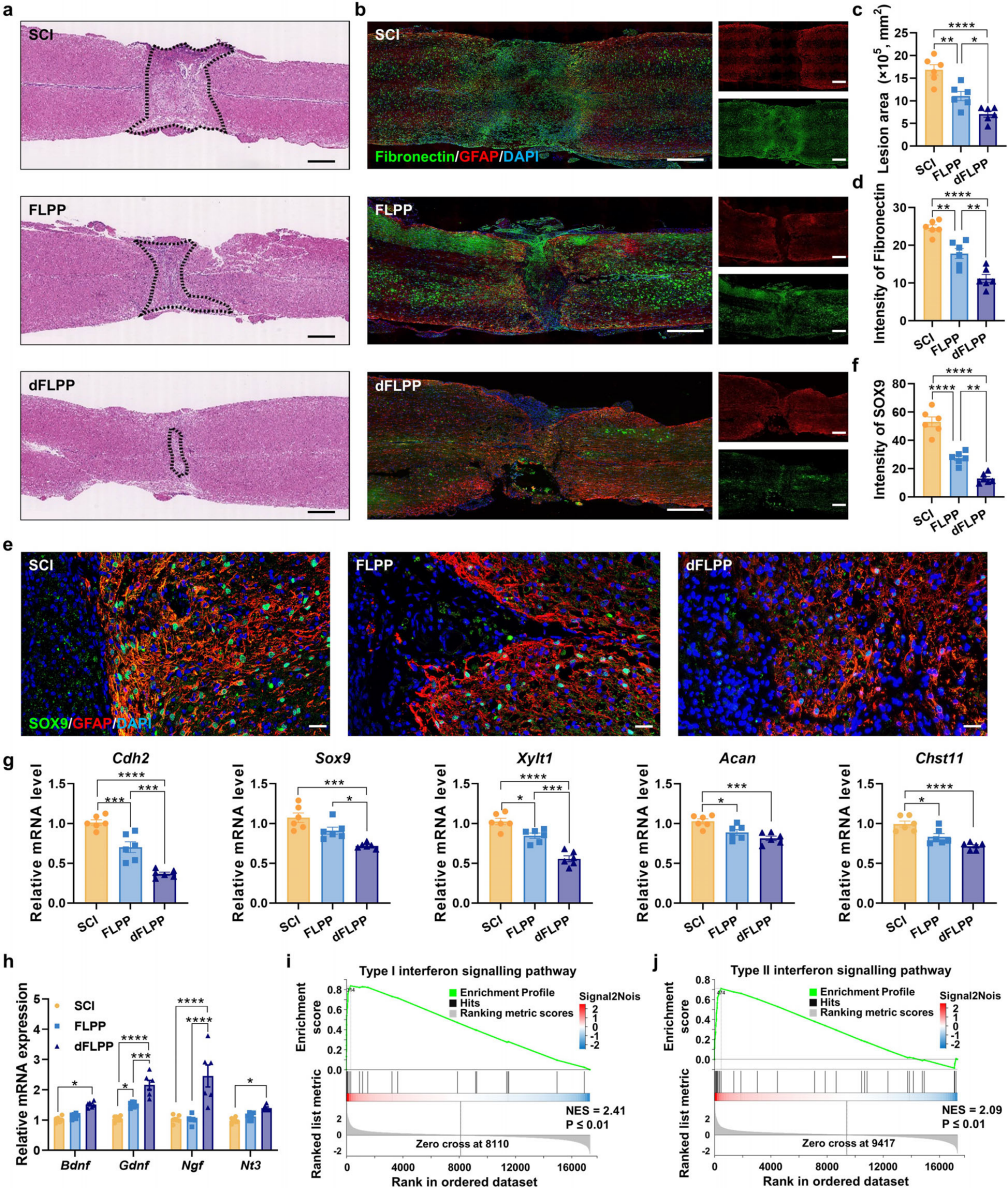
dFLPP promotes scar‐free healing and astrocyte reprogramming following SCI. a) H&E staining of spinal cord sections from SCI, FLPP, and dFLPP groups at 28 dpi. Scale bar: 500 µm. b) Immunofluorescent staining of fibronectin (green), co‐stained with GFAP (red), and nuclei (blue) at 28 dpi. Scale bar: 500 µm. c) Quantification of lesion area based on H&E‐stained sections across treatment groups (*n* = 6). d) Relative fluorescence intensity of fibronectin staining in each group (*n* = 6). e,f) Immunofluorescence staining (e) and quantification (f) of SOX9^+^ astrocytes in spinal cord lesions at 28 dpi (*n* = 6). Scale bar: 20 µm. g) Relative mRNA expression of SA markers (*Cdh2*, *Sox9*, *Xylt1*, *Acan*, *Chst11*) in the lesioned spinal cord at 28 dpi (*n* = 6). h) Relative mRNA expression of neurotrophic and regenerative genes (*Bdnf*, *Gdnf*, *Ngf*, *Nt3*) at 7 dpi (*n *= 6). i,j) GSEA showing activation of type I (i) and type II (j) interferon signaling pathways in PAs treated with CSPGs (3.34 µg mL^−1^) in combination with dFLPP (2 µg mL^−1^ pChABC) for 72 h in vitro, compared to CSPG treatment alone (*n* = 3). Data are mean ± SEM. Statistical comparisons used one‐way ANOVA with Tukey's post hoc test (≥3 groups). *p < 0.05, **p < 0.01, ***p < 0.001, ****p < 0.0001.

The reciprocal crosstalk between astrocytes and microglia is pivotal in orchestrating glial scar formation after SCI. Having demonstrated that dFLPP reduces CSPG accumulation and reprogrammes the microglial phenotype, we next investigated whether these effects led to diminished SA differentiation and attenuated fibrotic scarring in vivo (Figure 6a). At 28 dpi, haematoxylin and eosin (H&E) staining revealed extensive fibrosis and significant disruption of parenchymal architecture in both untreated and FLPP‐treated SCI mice. In contrast, dFLPP‐treated animals exhibited markedly reduced fibrotic density and better‐preserved parenchymal organization (**Figure** **7**a,b). Immunostaining for fibronectin, a major ECM component enriched in glial scars, corroborated these observations; intense fibronectin deposition was evident in the control groups, whereas the dFLPP group displayed a substantial reduction (Figure 7c,d). These findings suggest that CSPG‐targeted degradation, combined with immune reprogramming via dFLPP, synergistically inhibits fibrotic ECM deposition and limits scar expansion.

To determine whether the reduction in fibrosis was associated with altered astrocyte fate, we conducted co‐immunostaining for GFAP and SOX9. In both the lesion core and penumbra of untreated and FLPP‐treated mice, abundant SOX9^+^ astrocytes were observed, indicating widespread SA differentiation. In contrast, dFLPP treatment significantly decreased the number of SOX9^+^ astrocytes (Figure 7e,f), suggesting impaired SA conversion. Transcriptomic analysis further revealed a marked downregulation of SA‐associated genes, including *Cdh2*, *Sox9*, *Xylt1*, *Acan*, and *Chst11* (Figure 7g). These genes are implicated in CSPG biosynthesis and scar stabilization; their suppression indicates reduced fibrotic programming and ECM production. Collectively, these results suggest that dFLPP interrupts the pathological astrocyte‐microglia feedback loop that promotes chronic scar formation.

Concurrently, we observed increased expression of RA‐associated genes, including *Nestin*, *Plaur*, *Ctnnb1*, *Mmp2*, and *Mmp9*, at 28 dpi in dFLPP‐treated mice (Figure S21, Supporting Information). RAs can preserve tissue integrity and support neuronal survival by secreting neurotrophic factors.^[^ ^64^ ^]^ In line with this role, the expression of neurotrophic genes, including brain‐derived neurotrophic factor (*Bdnf*), glial cell line‐derived neurotrophic factor (*Gdnf*), nerve growth factor (*Ngf*), and neurotrophin‐3 (*Nt3*), was significantly upregulated in the spinal cords of dFLPP‐treated mice at 7 dpi (Figure 7h). These neurotrophic factors are essential for neuronal viability, axonal regeneration, and synaptic remodeling, suggesting that dFLPP promotes a neuroregenerative astrocyte phenotype in parallel with scar suppression.

To further elucidate the transcriptional mechanisms underlying this phenotypic reprogramming, we performed bulk RNA sequencing followed by pathway enrichment analysis. GO mapping identified the activation of immune‐regulatory pathways, particularly those involving interferon (IFN) signaling (Figure S22, Supporting Information). GSEA confirmed significant upregulation of both Type I and Type II IFN response signatures in dFLPP‐treated spinal cords (Figure 7i,j). IFN signaling has been previously implicated in regulatory astrocyte states that contribute to inflammation resolution and immune containment.^[^ ^65^ ^]^ These results demonstrate that dFLPP also drives astrocytic reprogramming toward a dual neurotrophic and immunoregulatory state, establishing a transcriptional landscape favorable for scar‐free repair and functional recovery.

### 2.7 dFLPP Promotes Neuronal Regeneration and Restores Locomotor Function after SCI

**Figure 8:**
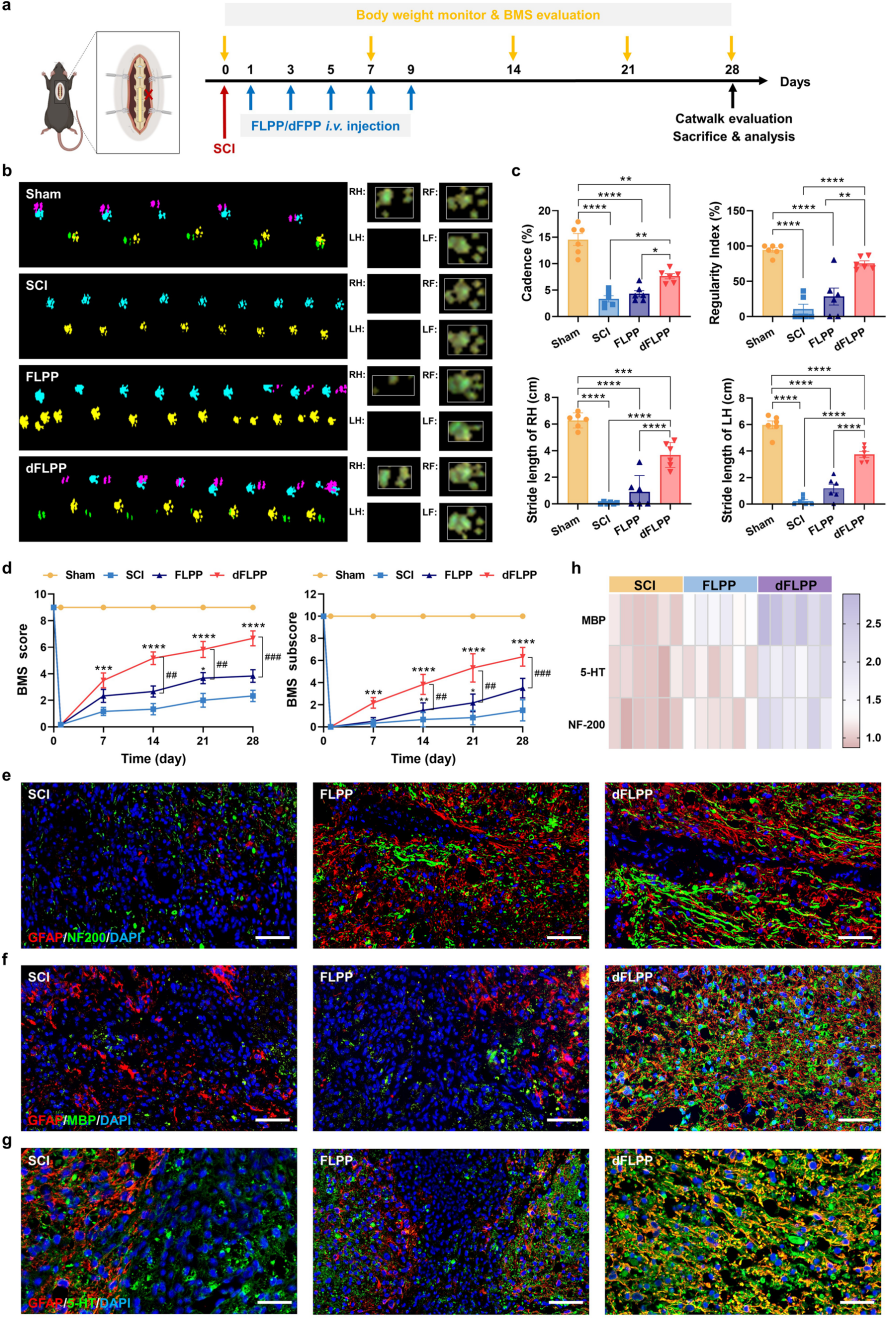
dFLPP treatment promotes functional recovery and neural regeneration following SCI. a) Schematic overview of the experimental timeline. FLPP or dFLPP was *i.v*. administered at 1 dpi every other day for five doses; pChABC dose, 0.5 mg kg^−1^. b) Representative footprint patterns captured by CatWalk gait analysis at 28 dpi in SCI, FLPP, and dFLPP‐treated mice (*n* = 6). c) Quantitative analysis of gait parameters derived from CatWalk, including cadence, regularity index, and stride lengths of the right (RH) and left (LH) hindlimbs (*n* = 6). d) BMS main scores and subscores tracking motor function recovery across 28 dpi (*n* = 6). e–g) Immunofluorescence staining of NF200 (e), MBP (f), and 5‐HT (g) in the lesioned spinal cord at 28 dpi. GFAP (red), NF200/MBP/5‐HT (green), and nuclei (DAPI; blue). Scale bar: 50 µm. h) Heatmap quantification of relative fluorescence intensity for NF200, MBP, and 5‐HT across treatment groups (*n* = 6). Data are mean ± SEM. Statistical comparisons used one‐way ANOVA with Tukey's post hoc test (≥3 groups).*p < 0.05, **p < 0.01, ***p < 0.001, ****p < 0.0001, ^##^ *p* < 0.01, ^###^ *p* < 0.001.

To evaluate the therapeutic efficacy of dFLPP in promoting functional recovery after SCI, we conducted a comprehensive assessment integrating behavioral, histological, and molecular analyses. Gait performance was evaluated using the CatWalk system at 28 dpi (**Figure** **8**a). Mice in the untreated SCI group exhibited severe locomotor deficits, characterized by complete loss of hindlimb stepping. FLPP‐treated mice showed partial recovery, with occasional placement of the left hindlimb. In contrast, dFLPP‐treated mice displayed near‐complete restoration of gait, demonstrating continuous, symmetrical, and well‐coordinated hindlimb movements that closely resembled those of uninjured controls (Figure 8b). Quantitative analysis of CatWalk parameters, including cadence, regularity index, and stride length, confirmed significant improvements in gait symmetry and interlimb coordination in dFLPP‐treated mice compared to both the SCI and FLPP control groups (Figure 8c).

These locomotor improvements were corroborated by longitudinal behavioral monitoring using the Basso Mouse Scale (BMS). From 7 dpi onward, dFLPP‐treated mice exhibited significantly higher BMS scores, with sustained increases in both main and sub‐scores throughout the 4‐week observation period (Figure 8d). The functional recovery in these animals was marked by an earlier return of weight‐supported plantar stepping and a gradual restoration of coordinated forelimb–hindlimb movements (Figure S23, Supporting Information), indicating the reactivation of spinal central pattern generator (CPG) circuits and the recovery of descending supraspinal control.

SCI commonly results in chronic autonomic dysfunction, such as neurogenic bladder, due to the disruption of visceral motor pathways, leading to impaired voiding reflexes, detrusor overactivity, and compensatory bladder wall hypertrophy. In our model, dFLPP‐treated mice exhibited markedly improved bladder histology, with reduced wall thickening and preserved structural organization, features absent in the SCI or FLPP control groups (Figure S24, Supporting Information). The restoration of spontaneous micturition in dFLPP‐treated mice further suggested the reactivation of spinal autonomic circuits. These findings demonstrate that, in addition to promoting motor recovery, dFLPP alleviates autonomic dysfunction by restoring visceral motor control. dFLPP also showed a favorable safety profile. No histopathological abnormalities were detected in major organs (Figure S24, Supporting Information), and body weight remained stable across all treatment groups (Figure S25, Supporting Information). Moreover, serum biomarkers of cardiac (creatine kinase and lactate dehydrogenase), hepatic (aspartate aminotransferase and alanine aminotransferase), and renal (serum creatinine and urea) function were within physiological ranges and not significantly altered (Figure S26, Supporting Information), supporting the good biosafety of dFLPP treatment.

To investigate the neurobiological basis of these functional improvements, we assessed markers associated with axonal integrity, myelination, and circuit plasticity. Immunostaining revealed a significant increase in the expression of neurofilament 200 (NF200) and myelin basic protein (MBP) in dFLPP‐treated spinal cords (Figure 8e,f,h), indicative of enhanced axonal preservation and remyelination, respectively. Serotonergic fiber density, visualized via 5‐hydroxytryptamine (5‐HT) staining, was also markedly elevated in the dFLPP group (Figure 8g,h). Given that descending serotonergic projections are critical for CPG activation and locomotor control, their restoration likely underpins the improved gait observed in behavioral assays. Furthermore, expression levels of oligodendrocyte transcription factor 2 (Olig2) were elevated in dFLPP‐treated tissues (Figure S27, Supporting Information), suggesting enhanced endogenous oligodendrogenesis, a key process for stabilizing regenerating axons and restoring conduction velocity through remyelination. Taken together, these results indicate that dFLPP promotes a comprehensive regenerative response involving axonal growth, myelin repair, reactivation of serotonergic circuits, and restoration of autonomic pathways. This coordinated mechanism culminates in substantial and functionally relevant recovery following SCI.

## 3 Discussion

Glial cells engage in a dynamic network of paracrine signaling that governs their phenotypic and functional states in response to CNS injury. Following traumatic SCI, this communication network undergoes pathological rewiring: ECM remodeling and glial reactivity converge to form a dense, CSPG‐rich scar that traps the cord in chronic inflammation and obstructs axon regeneration. While CSPGs have traditionally been viewed as physical barriers to neurite extension, emerging evidence, including our own, suggests that they act as active metabolic modulators within the glial niche. In this study, we demonstrate that CSPGs function as upstream modulators that suppress the CYP450 detoxification pathway in microglia. This suppression destabilizes microglial homeostasis, amplifies the secretion of pro‐inflammatory cytokines, and consequently induces neighboring RAs to adopt an SA phenotype. The subsequent increase in CSPG production by SAs further activates microglia, establishing a self‐perpetuating feed‐forward loop that maintains chronic inflammation and fibrotic scarring in the spinal cord. By targeting this pathological circuit with a ROS‐responsive, CTGF‐binding nanoplatform (dFLPP) designed to specifically enhance ChABC expression at the RA‐rich lesion border, we successfully interrupt this cascade, promoting both locomotor and autonomic functional recovery.

Previous studies have demonstrated that CSPGs activate macrophages through TLR4 and that microglia play a crucial role in scar formation.^[^ ^12^, ^46^ ^]^ In this study, we bridge these concepts by demonstrating that CSPG‐mediated inhibition of CYP450 functions as a “metabolic brake”, locking microglia into a persistent pro‐inflammatory state. This metabolic reprogramming impedes the resolution of inflammation and propagates astrocytic fibrosis. Pharmacological inhibition of CYP450 mimics the microglial phenotype induced by CSPGs, thereby identifying this pathway as a mechanistic node. These insights position the interplay between ECM signaling and intracellular metabolism as a viable therapeutic axis in SCI. Functionally, dFLPP‐mediated degradation of CSPGs restored CYP450 gene expression, reduced pro‐inflammatory cytokine production in microglia, and reinstated immunological homeostasis. By targeting delivery to the lesion border during the subacute phase, dFLPP minimizes off‐target effects while potentially preserving the acute protective functions of CSPGs in injury containment.^[^ ^13^ ^]^ Furthermore, dFLPP modulated astrocyte phenotypes, suppressing SA differentiation while enhancing the production of neurotrophic factors from RAs. We also observed that dFLPP treatment activated IFN‐associated transcriptional programmes in astrocytes, which have been linked to inflammation resolution without compromising immune surveillance.^[^ ^66^ ^]^ These dual effects on ECM remodeling and glial plasticity create a regenerative microenvironment.^[^ ^65^, ^66^ ^]^

Although our study centers on SCI, the CSPG‐glial interaction we describe is likely relevant to other CNS disorders marked by chronic neuroinflammation. CSPG accumulation is a hallmark of traumatic brain injury, ischaemic stroke, and neurodegenerative disease,^[^ ^67^, ^68^, ^69^ ^]^ where it coincides with inhibitory ECM remodeling, heightened glial reactivity, and persistent inflammation.^[^ ^70^, ^71^, ^72^ ^]^ Our data extend this view by showing that CSPGs actively reprogram innate immune cells via metabolic suppression, delineating a CSPG‐microglia‐astrocyte feed‐forward loop that may underlie glial dysfunction beyond SCI. Given that CSPGs exert stage‐dependent effects, our strategy emphasizes spatiotemporal precision in CSPG editing. dFLPP restricts ChABC expression to CTGF‐enriched RAs and employs ROS‐gated release, enabling targeted intervention during the subacute window when scar‐associated programs are consolidating, while sparing intact parenchyma and preserving early protective functions of CSPGs. Its modular design makes the platform adaptable to other proteoglycan‐rich pathologies. Nevertheless, several limitations warrant consideration. Our experiments were conducted in a single thoracic contusion model in mice and focused on a subacute intervention window. The efficacy of dFLPP remains to be determined in other SCI scenarios, such as cervical lesions, lateral hemisections, or chronic scars, where variations in CTGF expression, ROS levels, or microglial states could influence targeting fidelity and therapeutic outcomes. Future studies should evaluate dFLPP performance across diverse spinal levels, injury severities, and timepoints. Moreover, combining dFLPP with pro‐regenerative strategies, such as axon guidance molecules or cell transplantation, could potentially enhance functional recovery. In summary, this study reveals a pathological feedback loop in which CSPG‐driven metabolic suppression sustains glial scarring and establishes controlled ChABC delivery as a powerful tool to precisely edit the injury microenvironment for effective scar‐free SCI repair.

## Conflict of Interest

The authors declare no conflict of interest.

## Supporting information



Supporting Information

## Data Availability

The data that support the findings of this study are available in the supplementary material of this article.
