# Supplementary material for: Disrupting CSPG‐Driven Microglia–Astrocyte Crosstalk Enables Scar‐Free Repair in Spinal Cord Injury
Source: Adv Sci (Weinh). 2025 Nov 12;13(6):e16269. doi: 10.1002/advs.202516269 (PMC12866793; doi:10.1002/advs.202516269)
Supplement: Supplementary file 1 — Supporting Information [file ADVS-13-e16269-s001.docx]

Supporting Information

**Disrupting CSPG-Driven Microglia-Astrocyte Crosstalk Enables Scar-free Repair in Spinal Cord Injury**

Yufei Zheng,^†^ Zhaowei Zhang,^†^ Zezhou Fu,^†^ Qingqing Wang, Siwen Zhang, Tingyu Zhang, Meifei Zhu, Shunwu Fan,^*^ Youqing Shen,^*^ Jiajia Xiang,^*^ and Xin Liu^*^

Y. Zheng, Z. Zhang, Z. Fu, Q. Wang, T. Zhang, S. Fan, X. Liu

Department of Orthopaedic Surgery, Sir Run Run Shaw Hospital, Zhejiang University School of Medicine, Hangzhou, Zhejiang 310016, China

Key Laboratory of Mechanism Research and Precision Repair of Orthopaedic Trauma and Aging Diseases of Zhejiang Province, Hangzhou, Zhejiang 310016, China

E-mail addresses: shunwu_fan@zju.edu.cn (S. Fan), xinliuzju@zju.edu.cn (X. Liu)

S. Zhang, Y. Shen, J. Xiang

Zhejiang Key Laboratory of Smart Biomaterials and Center for Bionanoengineering, College of Chemical and Biological Engineering, Zhejiang University, Hangzhou, Zhejiang 310027, China

E-mail addresses: shenyq@zju.edu.cn (Y. Shen), xiang_jj@zju.edu.cn (J. Xiang)

M. Zhu

College of Animal Sciences, Zhejiang University, Hangzhou 310058, China

† These authors contributed equally to this work.

Materials and methods

Materials

B-PDEA was synthesised as previously described ^[1, 2]^. Branched PEI (25 kDa) was purchased from Sigma-Aldrich (Shanghai, China). CHEMS was purchased from Tokyo Chemical Industry Co., Ltd. (Shanghai, China). DOPE and DSPE-PEG were purchased from AVT Pharmaceutical Tech Co., Ltd. (Shanghai, China). DAG peptide was purchased from Sangon Biotech Co., Ltd. (Shanghai, China). CSPGs were sourced from Millipore (CA, USA). Bergamottin and ChABC were obtained from MedChemExpress (NJ, USA). ChABC plasmid, Cyp1a1 plasmid, pGL4.13 luciferase plasmid, and EGFP plasmid were obtained from GenePharma (Suzhou, China). The Luciferase assay system was purchased from Promega (WI, USA). LysoTracker Green, Hoechst 33342, Lipofectamine^TM^ 3000, and DAPI were purchased from Thermo Fisher Scientific (Waltham, MA, USA). Cy5-labeled DNA was prepared using the Label IT® Nucleic Acid Labeling Kit (Mirus Bio, WI, USA). LPS and the bicinchoninic acid (BCA) protein assay kit were purchased from Beyotime Biotechnology (Shanghai, China). The ELISA kits were purchased from Jingmei Biotechnology (Jiangsu, China). Total RNA was extracted using the SteadyPure RNA Extraction Kit (Accurate Biotechnology Co., Ltd, Hunan, China).

## Methods

### Primary cell isolation

Primary glial cells were isolated from the cerebral cortices of neonatal mice (postnatal day 1) following institutional ethical guidelines. Neonatal mice were euthanized, and their brains were rapidly extracted and transferred to ice-cold calcium- and magnesium-free Hank's Balanced Salt Solution (HBSS). The cortices were dissected, and the meninges were carefully removed by microdissection under a stereomicroscope. The cortical tissues were then minced and digested with trypsin at 37 ℃ for 15 min. DNase I (final concentration: 0.1 mg/mL) was added to prevent cell clumping. After neutralization with Dulbecco's Modified Eagle Medium (DMEM) supplemented with 10% fetal bovine serum (FBS), the resulting cell suspension was passed through a 70 μm cell strainer and centrifuged at 300 × g for 5 min. The cell pellet was resuspended in FBS-containing DMEM and seeded into poly-L-lysine-coated culture flasks at a density of 1 × 10^6^ cells/mL. Cultures were maintained at 37 ℃ in a humidified atmosphere of 5% CO_2_, with medium replaced every 2 days to remove debris and non-adherent cells.

When cell confluency reached approximately 90% after 7-10 days, astrocytes were enriched by shaking the flasks at 250 rpm for 3 h at 37 ℃ to detach loosely adherent microglia and OPCs. The remaining adherent astrocytes were washed with fresh medium, trypsinized, and replated into new poly-L-lysine-coated flasks. Astrocytes were cultured in DMEM supplemented with 10% FBS and 1% penicillin-streptomycin. To isolate microglia, the flasks were shaken at 180 rpm for 1 h at 37 ℃. The supernatant containing detached microglia was collected and centrifuged at 300 × g for 5 min. The pellet was resuspended in a fresh DMEM with 10% FBS and plated into poly-L-lysine-coated culture dishes. The cultures were maintained by changing the medium every 2-3 days. All primary cells were used for experiments once they reached 80-90% confluency.

To induce a reactive phenotype, PAs were stimulated with LPS at a final concentration of 1 mg/mL in a complete medium for 18 h in T25 flasks as previously reported ^[3]^. The RAs were then used for further experiments.

### P450 inhibition in microglia

BV2 cells were seeded onto a 12-well plate at a density of 1 × 10^6^ cells per well and incubated for 24 h to allow adherence. Then, the cells were stimulated with LPS (500 ng/mL) according to the previous study ^[4]^, in the presence or absence of P450 inhibitor (bergamottin, 10 μM) for an additional 24 h. Post-treatment, the cells were harvested and processed for quantitative real-time PCR (qRT-PCR) analysis to analyze the expression of genes associated with inflammatory responses.

### P450 restoration in microglia

BV2 cells were seeded onto a 12-well plate at a density of 1 × 10^6^ cells per well and incubated for 24 h to allow adherence. Cells were then transfected with a *Cyp1a1* overexpression plasmid using Lipofectamine^TM^ 3000 according to the manufacturer's protocol. After transfection, both control and *Cyp1a1*-overexpressing BV2 cells were treated with CSPGs (3.34 μg/mL) for 48 h. Following treatment, total RNA was extracted for qRT-PCR analysis.

### Assessment of TLR4 involvement in CSPG-induced CYP450 suppression

To investigate the role of TLR4 signaling in the regulation of CYP450-related gene expression, BV2 microglial cells were seeded in 12-well plates at a density of 1 × 10^6^ cells/well. Cells were then treated under three conditions: (1) untreated control, (2) CSPG stimulation (3.34 μg/mL), and (3) co-treatment with CSPGs and the selective TLR4 inhibitor TAK-242 (50 nM) as previously reported ^[5]^. After 24 h of incubation, cells were harvested, and total RNA was extracted for qRT-PCR analysis.

### Preparation and characterization of dFLPP

DSPE-PEG-DAG was synthesised *via* copper-catalyzed azide-alkyne cycloaddition by reacting the alkyne-functionalized DAG peptide with DSPE-PEG-N_3_ at a molar ratio of 1.2:1. The resulting conjugate was purified by dialysis and lyophilized for storage. To prepare the fusogenic lipid envelope, lipid components, including DOPE, DSPE-PEG-DAG, and CHEMS, were dissolved in chloroform at a molar ratio of 7.65:1.35:2. The organic solvent was removed by rotary evaporation to form a thin lipid film, which was subsequently hydrated overnight in 10 mM HEPES buffer (pH 7.4) at room temperature. The lipid suspension was then ultrasonicated for 10 min and filtered through a 0.22 µm membrane (Millipore). B-PDEA was dissolved in 10 mM HEPES buffer (pH 7.4) and mixed with plasmid DNA (40 μg/mL) at a 1:1 volume ratio based on the desired N/P ratio, then vortexed to form polyplexes. dFLPP was assembled by incubating the liposome solution with B-PDEA/pDNA polyplexes at room temperature overnight. The resulting formulation was characterized by DLS to determine the hydrodynamic diameter and zeta potential. Particle morphology was observed by cryo-TEM.

### ROS-responsive DNA release of dFLPP

To evaluate the ROS responsiveness of the dFLPP delivery system, 25 μL of dFLPP containing pChABC (N/P ratio = 10) was incubated with hydrogen peroxide (H_2_O_2_) at final concentrations of 0, 0.5, 1, 2, 5, and 10 mM for 1 h at 37 ℃. Following incubation, the samples were loaded onto a 0.8% agarose gel containing GelRed (Biotium) for nucleic acid staining and subjected to electrophoresis in 1× TAE buffer at 100 V for 30 min using a GT Mini-Gel Casting System (Bio-Rad). For reference, 10 μL of free plasmid DNA was similarly incubated with or without 10 mM H_2_O_2_ and then electrophoresed under identical conditions.

### Cellular uptake and subcellular distribution

To evaluate cellular uptake and subcellular distribution of dFLPP, PAs were seeded onto confocal-compatible culture plates at a density of 8×10^4^ cells per well and stimulated with 1 μg/mL LPS for 24 h to induce an RA phenotype. For confocal imaging, RAs were incubated with dFLPP or FLPP formulations at a ^Cy5^DNA dose of 1 μg/mL in a serum-free medium for 1 h at 37 ℃. Following incubation, cells were stained with Hoechst 33342 (2 drops per well, 15 min), washed with PBS, and imaged using confocal laser scanning microscopy (CLSM; Zeiss, LSM 880).

For quantitative analysis of cellular uptake, RAs were treated under the same conditions with dFLPP or FLPP formulations at a ^Cy5^DNA dose of 100 ng/mL for 1 h in serum-free medium. After incubation, cells were washed with cold PBS containing 1% bovine serum albumin (BSA), isolated, and resuspended in PBS containing 1% BSA. Cy5 fluorescence intensity was measured using flow cytometry (Beckman, CytoFLEX), and the percentage of Cy5-positive cells was calculated using FlowJo software (v10).

To investigate subcellular localization, RAs were incubated with DiI-labeled dFLPP at a ^Cy5^DNA dose of 1 μg/mL and a DiI dose of 0.05 μmol/μg DNA for 4 h in a serum-free medium. Following this, the cells were washed, incubated in complete growth medium for an additional 18 h, and stained with LysoTracker Green (200 nM, 20 min) and Hoechst 33342 (2 drops, 15 min). Cells were then imaged by CLSM to visualize the co-localization of ^Cy5^DNA, DiI-labeled lipid, and lysosomes.

To assess cell-type selectivity in a heterogeneous astrocyte population, PAs and RAs were mixed at a ratio of 1:1 and incubated with dFLPP at a ^Cy5^DNA dose of 100 ng/mL in a serum-free medium for 1 h at 37 ℃. Post-incubation, cells were washed with PBS containing 1% BSA and blocked in PBS containing 5% BSA for 0.5 h. Cells were then stained with an anti-CTGF primary antibody followed by a FITC-conjugated secondary antibody, washed, and resuspended in cold PBS containing 1% BSA. Flow cytometry was used to quantify the proportion of Cy5-positive cells and the intensity in CTGF-positive (CTGF^+^) and CTGF-negative (CTGF^-^) subpopulations.

To evaluate the correlation between CTGF expression and dFLPP uptake, individual astrocytes within the mixed population were co-analyzed for FITC fluorescence (reflecting CTGF levels) and intracellular Cy5 fluorescence (indicating dFLPP uptake). Cells were stratified into seven subpopulations based on FITC intensity, and the mean Cy5 signal was quantified for each subgroup to assess the relationship between CTGF expression and dFLPP internalization.

### *In vitro* gene transfection

*In vitro* transfection efficiency was evaluated by quantifying luciferase expression following the delivery of pLuc plasmid DNA. PAs and BV2 cells (1 × 10^4^ cells/well) were seeded in 96-well plates and transfected with B-PDEA polyplexes at a pLuc dose of 2 μg/mL in serum-free medium for 4 h. The medium was then replaced with a complete culture medium, and the cells were cultured for an additional 48 h. Luciferase activity was measured using a luciferase assay kit following the manufacturer's instructions. The total protein content in each well was measured using a BCA protein assay kit to normalize luciferase activity.

PAs were seeded at 5 × 10^4^ cells per well in 12-well plates for qRT-PCR and at 1 × 10^4^ cells per well in 6-well plates for western blotting. Cells were transfected with FLPP or dFLPP containing pChABC at a DNA concentration of 2 μg/mL. After 48 h, total RNA was extracted and subjected to qRT-PCR, and protein levels were analyzed by Western blotting using standard protocols.

### Transwell co-culture system

To investigate microglia-astrocyte interactions, a Transwell co-culture system (12-well format, 0.4  μm pore size; Corning) was employed. PM (1 × 10^5^ cells/well), either pre-treated with 3.34 μg/mL CSPGs for 24 h or left untreated, were seeded into the upper chamber inserts. PAs (5×10^5^ cells/well) were plated in the corresponding lower chambers. After 48 h of co-incubation, astrocytes were harvested for downstream analyses. Phenotypic changes in astrocytes were assessed *via* flow cytometry and qRT-PCR. The levels of secreted CSPG family members, including brevican and neurocan, in the culture medium were quantified using ELISA kits (JM-13442M2 and JM-13438M2) following the manufacturer's instructions.

For flow cytometric detection of SOX9- or MMP2-positive astrocytes, astrocytes from the lower chambers were collected, washed twice with PBS, and permeabilized using 0.1% Triton X-100 in PBS for 15 min at room temperature. Cells were then washed with PBS supplemented with 1% BSA and 1 mM EDTA and blocked with PBS containing 5% BSA for 30 min. Subsequently, cells were stained with primary antibodies against SOX9 (Abcam, ab185230) or MMP2 (Proteintech, 2B10D1) for 30 min at 4 ℃, followed by FITC- or A647-conjugated secondary antibodies for 30 min in the dark. Samples were analyzed using a CytoFLEX LX flow cytometer (Beckman Coulter), and data were processed using CytExpert software. The gating strategy is shown in Figure S8b.

### Transcriptomic analysis

To assess the transcriptional impact of CSPG stimulation and its enzymatic degradation on astrocytes and microglia, PAs and PMs were seeded in 12-well plates at a density of 1×10^6^ cells per well. PAs were treated with CSPGs (3.34 µg/mL), either alone or in combination with dFLPP (2 μg/mL pChABC) for 72 h. PMs were cultured under three conditions: untreated (control), treated with CSPGs (3.34 µg/mL) alone, or co-treated with CSPGs and dFLPP (2 μg/mL pChABC) for 72h. After treatment, total RNA was extracted and subjected to RNA sequencing, which was performed by Huada Gene Technology Co., Ltd. (Wuhan, China). Raw and processed sequencing data were analyzed using the Dr. Tom platform (biosys.bgi.com), a cloud-based bioinformatics suite provided by Huada. In addition, publicly available RNA-seq data from Aldh1l1^+^ astrocytes at various post-injury time points were retrieved from the Gene Expression Omnibus (GEO) under accession number GSE241628.

### CSPG impact on microglia profile

To investigate the transcriptional impact of CSPG stimulation, BV2 cells were seeded onto a 12-well plate at a density of 1 × 10^6^ cells per well. Then, cells were treated with CSPGs (3.34 μg/mL) and CSPGs + dFLPP (2 μg/mL) for 72 h. After treatment, cells were harvested and processed for qRT-PCR analysis.

To evaluate the impact of CSPGs in a dose and time-dependent manner, BV2 cells were treated with CSPGs at different dosages (1.17, 3.34, or 6.68 μg/mL) for 48 h or with 3.34 μg/mL CSPG for various time periods (24, 48, or 72 h). Post-treatment, the cells were harvested and processed for qRT-PCR analysis to analyse the expression of genes associated with the CYP450 family.

### SCI model establishment

A contusive SCI model was established in mice under anaesthesia with intraperitoneal administration of sodium pentobarbital (40 mg/kg). The surgical site was shaved and disinfected, and a midline incision was made to expose the spinal column at the T9-T10 vertebral level. SCI was induced by applying a microvascular clip (Biemer Vessel Clip, 14687, Aesculap) bilaterally to the spinal cord for 1 min, ensuring consistent compression injury. In the sham group, the spinal cord was exposed without clip application. Following injury, the muscle and skin layers were closed using absorbable sutures. Post-operative care included close monitoring and manual bladder expression twice daily to prevent urinary retention. Motor function was evaluated at 1 day post-injury (dpi) using the BMS, and only mice scoring zero, indicating complete hindlimb paralysis, met the inclusion criteria and were used for further studies. All animal procedures were approved by the Animal Care and Use Committee of Zhejiang University (protocol number: ZJU20230062).

### *In vivo* biodistribution and transfection of dFLPP in SCI mice

To assess *in vivo* biodistribution, DiI-labeled dFLPP loaded with EGFP plasmids was intravenously (*i.v.*) administered into SCI mice at 1 dpi. Real-time imaging of DiI fluorescence was conducted at 4, 8, 12, and 24h post-injection using a Lumina III Imaging System (PerkinElmer, USA). At 24 h post-injection, three mice per group were sacrificed for *ex vivo* DiI imaging of major organs, including the liver, spleen, lungs, kidneys, heart, and spinal cord. At 48 h post-injection, an additional three mice per group were sacrificed for *ex vivo* GFP fluorescence imaging of organs or tissues to assess gene transfection efficiency. Spinal cords were then embedded in optimal cutting temperature (O.C.T.) compound and cryosectioned. Tissue sections were stained with propidium iodide (PI) to label nuclei and imaged to measure the transfection of dFLPP within the injured spinal cord.

To evaluate the cell-type targeting specificity of the delivery system *in vivo*, SCI mice were *i.v.* administered Rhodamine B-labeled dFLPP at 1 dpi (0.5 mg/kg pChABC). At 24 h post-injection, animals were euthanized, and spinal cords were promptly harvested. Tissues were either embedded in O.C.T. compound for cryosectioning or enzymatically dissociated into single-cell suspensions for flow cytometric analysis. For immunofluorescence staining, 10-μm-thick frozen sections were fixed, permeabilized, and incubated with primary antibodies against GFAP (an astrocyte marker) and CD31 (an endothelial marker), followed by fluorophore-conjugated secondary antibodies and DAPI for nuclear counterstaining. Fluorescent images were acquired using CLSM. For flow cytometric analysis, dissociated spinal cord cells were filtered through a 70-μm strainer, permeabilized with saponin, blocked with 5% BSA, and stained with anti-GFAP and anti-CD31 primary antibodies and corresponding fluorophore-conjugated secondary antibodies. The fluorescence intensities of Rhodamine B and CTGF within GFAP^+^ and CD31^+^ populations were quantified using flow cytometry and analyzed using FlowJo software. The gating strategy is shown in Figure S16d.

### *In vivo* assessment of SCI repair

Mice subjected to SCI were randomly allocated into three treatment groups: PBS, FLPP, or dFLPP (0.5 mg/kg pChABC). Treatments were *i.v.* administered every other day from 1 dpi for a total of five doses. Mice's body weight was recorded weekly.

Hindlimb motor function was evaluated using the BMS locomotor rating system at 0, 7, 14, 21, and 28 dpi. During each assessment, mice were placed individually in an open field and observed for 4 min by two independent assessors blinded to group assignments. The BMS score ranges from 0 (complete hindlimb paralysis) to 9 (normal locomotion with coordinated stepping, proper paw placement, trunk stability, and tail positioning). Parameters, including joint movement, paw placement, stepping ability, coordination, and trunk control, were assessed.

In addition, gait performance was analyzed using the CatWalk XT automated gait analysis system (Noldus Information Technology, Wageningen, The Netherlands). All animals were acclimatized to the system prior to baseline testing, which was conducted 3 days before SCI or sham surgery. For each trial, animals were placed at one end of a horizontal glass walkway and allowed to traverse freely towards the opposite end. Only spontaneous, uninterrupted runs were used for analysis. Gait parameters, including paw placement, cadence, regularity index, and stride length, were captured and processed using CatWalk XT software (version 10.5).

### Biological safety evaluation

After treatment, whole blood was collected from the Sham, SCI, FLPP, and dFLPP groups and processed for serum. Samples were allowed to clot, centrifuged, and the supernatant serum was aliquoted and stored at -80 ℃ until analysis. Serum biochemistry was performed at Calibra Laboratory, DIAN Diagnostics (Hangzhou), on a Roche cobas c701 automated clinical chemistry analyzer using commercial reagents from MikeBio, following the manufacturers' instructions.

### Haematoxylin and eosin (H&E) and immunofluorescence staining

Spinal cords from each treatment group were fixed in 4% paraformaldehyde (PFA) at room temperature for 24 h. Fixed tissues were embedded in paraffin and sectioned at 10 μm thickness. For H&E staining, sections were deparaffinized, rehydrated through a graded ethanol series to distilled water, stained with haematoxylin for 5 min, rinsed in running tap water, differentiated in 1% acid alcohol, and counterstained with eosin for 1 min. Following dehydration and clearing, slides were mounted with coverslips using a mounting medium.

For immunofluorescence, paraffin sections were deparaffinized and rehydrated. After antigen retrieval, sections were blocked with QuickBlock^TM^ Blocking Buffer for Immunol Staining (P0260, Beyotime) to prevent non-specific binding. Primary antibodies, diluted in QuickBlock^TM^ Primary Antibody Dilution Buffer for Immunol Staining (P0262, Beyotime), were applied and incubated overnight at 4 ℃. After washing, fluorophore-conjugated secondary antibodies, diluted in QuickBlock^TM^ Secondary Antibody Dilution Buffer for Immunofluorescence (P0265, Beyotime), were applied for 1 h at room temperature in the dark. Nuclei were counterstained with DAPI, and sections were mounted with an anti-fade medium.

Primary antibodies included: GFAP (Abcam, ab4674), NeuN (Abcam, ab177487), GAP-43 (Abcam, ab75810), NF-200 (Abcam, ab207176), Nestin (Santa Cruze, sc23927), ChAT (Abcam, ab181023), 5-HT (Abcam, ab254358), Olig2 (Abcam, ab109186), Iba1 (Abcam, ab283346), iNOS (Abcam, ab178945), Arg1 (Abcam, ab96183), SOX9 (Abcam, ab185966), NG2 (Abcam, ab275024), P2y12 (Invitrogen, 4H5L19), Fibronectin (Abcam, ab268020), CD31(Abcam, ab7388), and CTGF (Abacm, ab318148) as indicated in Table S2.

### mRNA and protein level analysis

Cells were washed with ice-cold PBS after different treatments before lysis. Total RNA was extracted from cultured cells using TRIzol reagent and purified with the SteadyPure RNA extraction kit (AG21024) according to the manufacturer's instructions. RNA concentration and purity were determined using a NanoDrop spectrophotometer. The samples were then subjected to qRT-PCR analysis. Primer sequences are listed in Supplementary Table 1.

For protein analysis, cells or tissue samples were lysed and processed for Western blotting as previously described ^[6]^. Protein concentrations were measured using the BCA Protein Assay Kit (P0012, Beyotime). Equal amounts of protein were separated by SDS-PAGE and transferred onto polyvinylidene difluoride (PVDF) membranes, which were blocked in tris-buffered saline with Tween-20 (TBST) containing 5% BSA and incubated with the indicated primary antibodies, followed by horseradish peroxidase (HRP)-conjugated secondary antibodies. Immunoreactive bands were visualized using an enhanced chemiluminescence (ECL) substrate and imaged using a VilBER imaging system.

Primary antibodies used for western blotting included ChABC (Abcam, ab256485), NG2 (Abcam, ab275024), Arg1 (Abcam, ab96183), iNOS (Abcam, ab178945), and P2Y12 (Invitrogen, 4H5L19) as indicated in Supplementary Table 2.

### Statistical analysis

Data are presented as mean ±  standard error of the mean (SEM). Statistical comparisons between two groups were performed using an unpaired, two-sided Student's t-test, while comparisons among multiple groups were conducted using one-way ANOVA. Analyses were carried out using GraphPad Prism (version 9.0). Statistical significance was defined as P < 0.05.

Supplementary figures

**
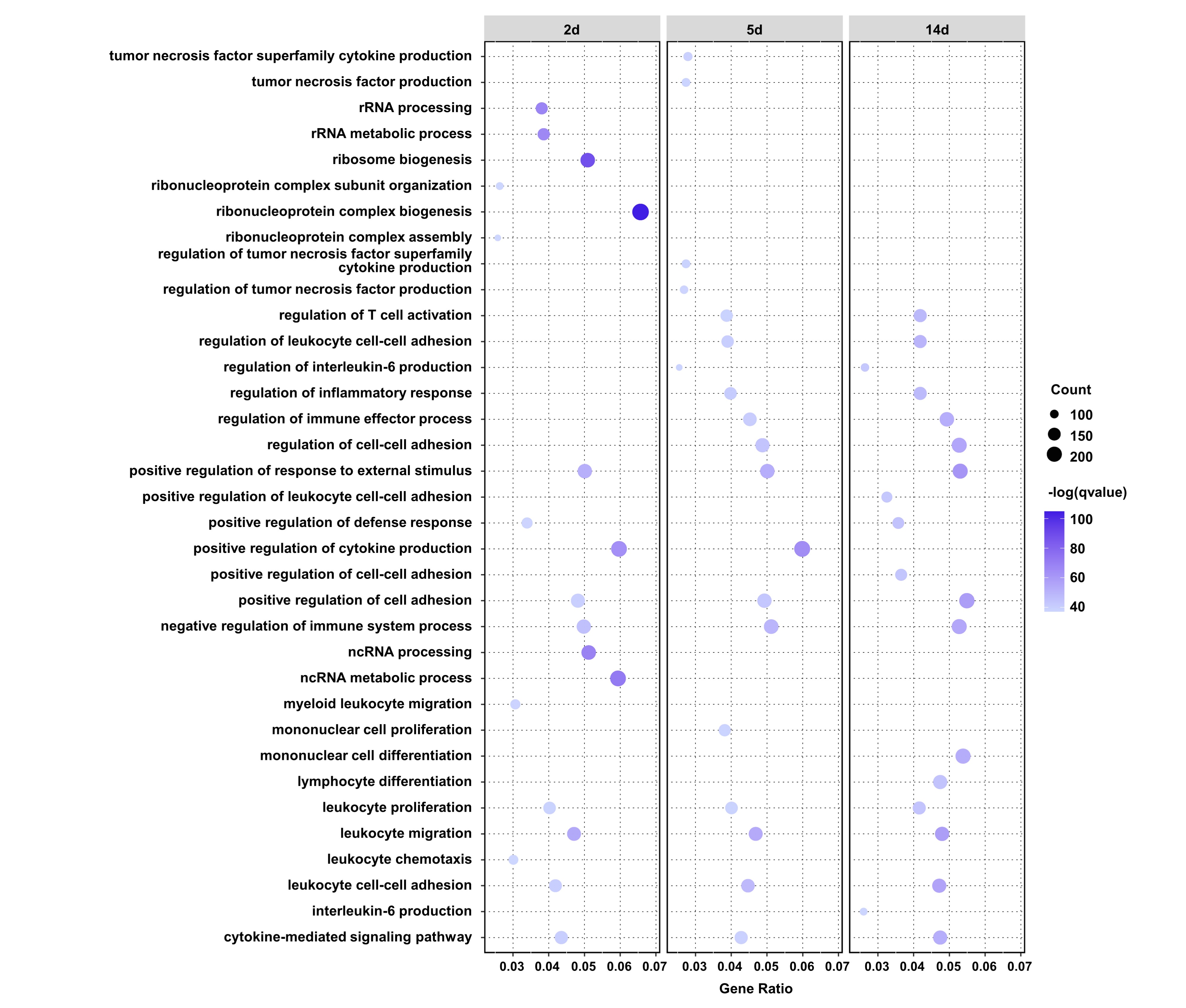
**

Figure S1. Gene Ontology (GO) enrichment analysis of the top upregulated differentially expressed genes (DEGs) in Aldh1l1^+^ astrocytes at 2, 5, and 14 days post-injury (dpi).


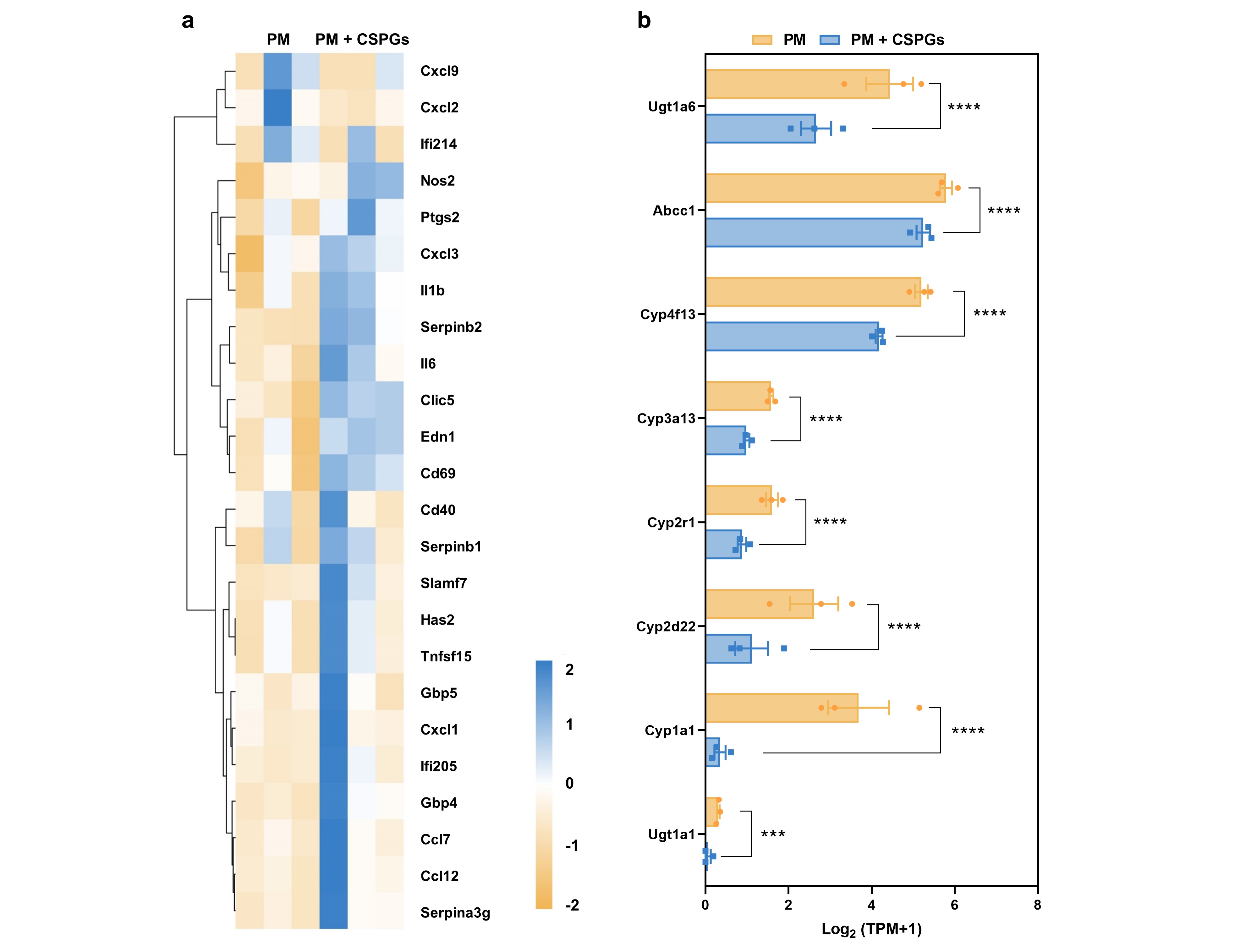


Figure S2. Transcriptomic profiling of CSPG-treated primary microglia (PM) showing upregulation of inflammatory genes (a) and downregulation of CYP450-associated genes (b). PMs were treated with or without CSPGs (3.34 μg/mL) for 72 h. Hierarchical clustering was performed using the Ward.D2 linkage method, and gene expression patterns were visualized as heatmaps (n = 3). Data are mean ± SEM. Statistical comparisons used unpaired two-tailed t-tests. ***p < 0.001, ****p < 0.0001.


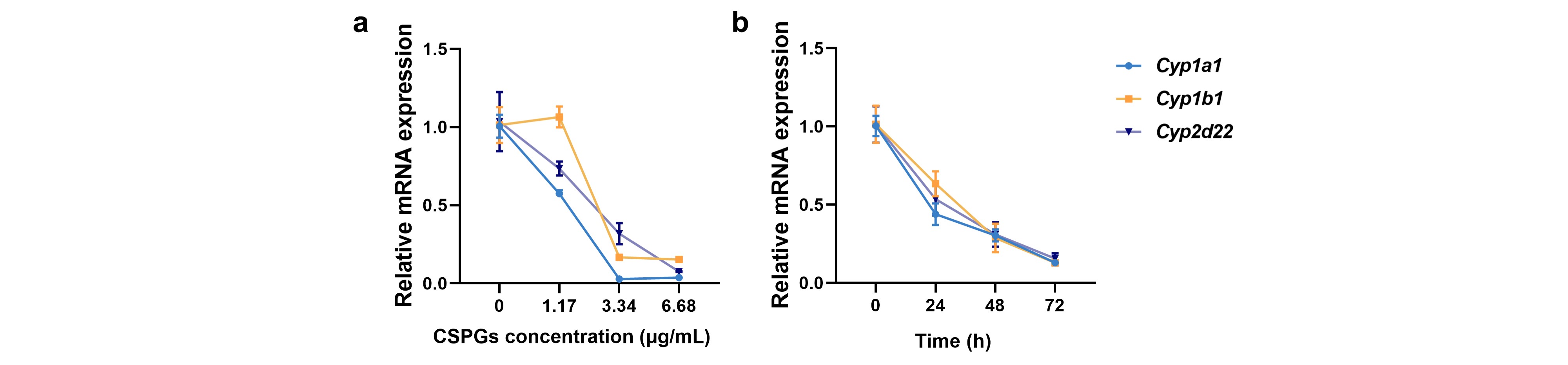


Figure S3. qRT-PCR analysis of mRNA levels of *Cyp1a1*, *Cyp1b1*, and *Cyp2d22* in BV2 cells treated at different CSPG concentrations (1.17, 3.34, or 6.68 μg/mL) for 48 h (a) or with CSPG (3.34 μg/mL) for varying time intervals (24, 48, or 72 h) (b) (n=3).


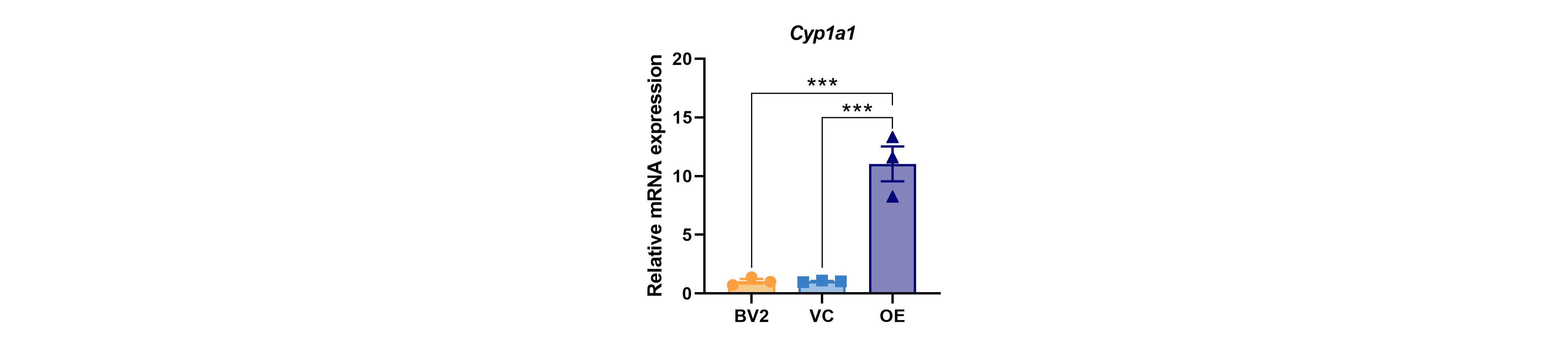


Figure S4. qPCR analysis of *Cyp1a1* expression levels in BV2 microglial cells. BV2 cells were transfected with either a *Cyp1a1* overexpression (OE) or vector control (VC) plasmid (1 μg/mL) using Lipofectamine 3000 for 48 h (n=3). Data are mean ± SEM. Statistical comparisons used one-way ANOVA with Tukey's post hoc test (≥ 3 groups). ***p < 0.001.


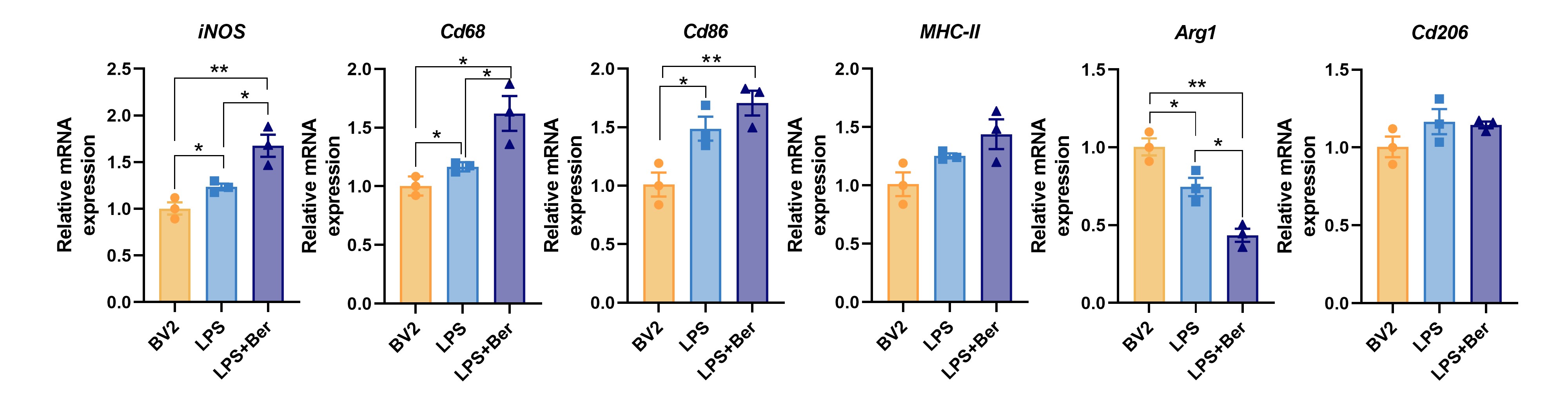


Figure S5. Quantitative RT-PCR analysis of mRNA expression levels of microglia phenotype-related genes in BV2 microglial cells stimulated with LPS (500 ng/mL), with or without bergamottin (Ber, 10 μM) for 24 h (n = 3). Data are mean ± SEM. Statistical comparisons used one-way ANOVA with Tukey's post hoc test (≥ 3 groups). *p < 0.05, **p < 0.01.


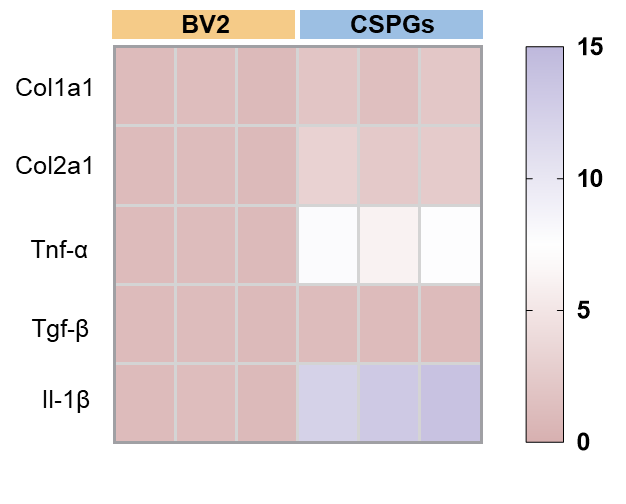


Figure S6. Quantitative RT-PCR analysis of mRNA expression levels of the indicated genes in BV2 microglial cells treated with or without CSPGs (3.34 μg/mL) for 24 h (n = 3).


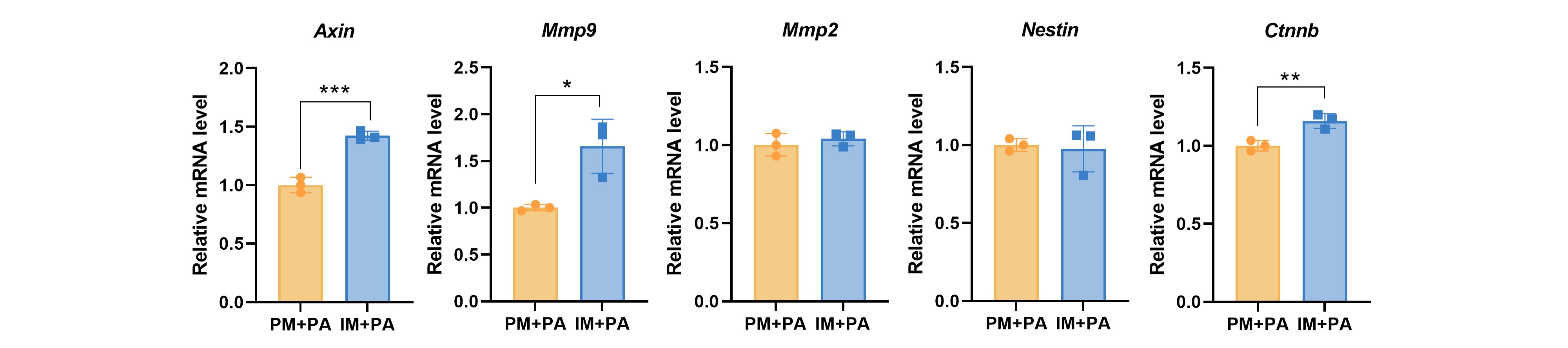


Figure S7. Quantitative RT-PCR analysis of mRNA expression levels of reactive astrocyte (RA)-associated markers in primary astrocytes (PAs) co-cultured for 48 h with either PMs or IMs (n = 3). IMs were induced by exposing PMs to CSPGs (3.34 μg/mL) for 24 h. Data are mean ± SEM. Statistical comparisons used unpaired two-tailed t-tests. *p < 0.05, **p < 0.01, ***p < 0.001.


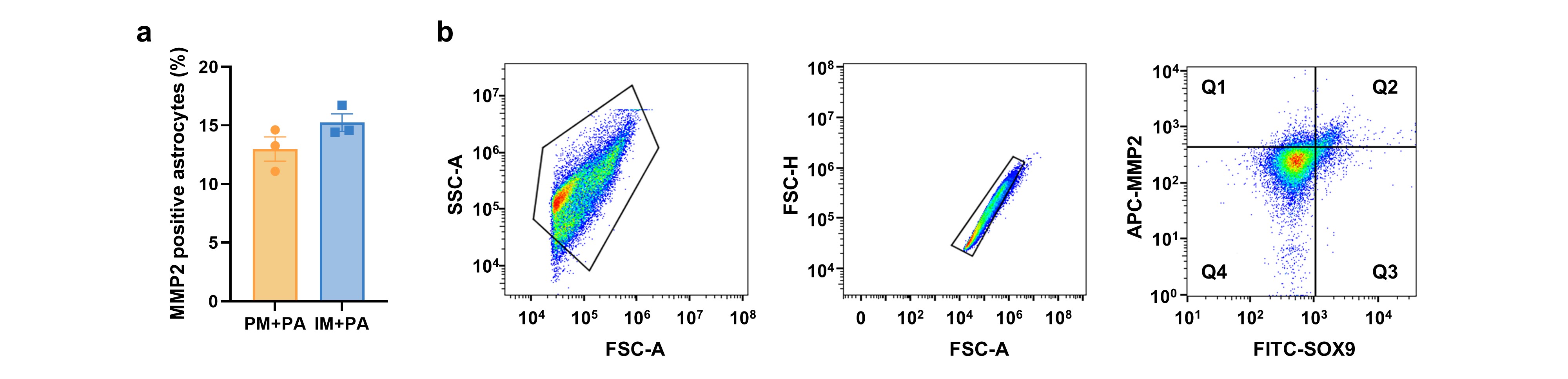


Figure S8. Flow cytometric analysis of astrocyte phenotypes induced by microglia. (a) Quantification of MMP2^+^ astrocytes (RAs) among total PAs from the lower chambers following 48 h co-culture with either unstimulated (PM) or CSPG-stimulated inflammatory microglia (IM) using a transwell co-culture system (n = 3). (b) Gating strategy used for astrocyte analysis by flow cytometry.


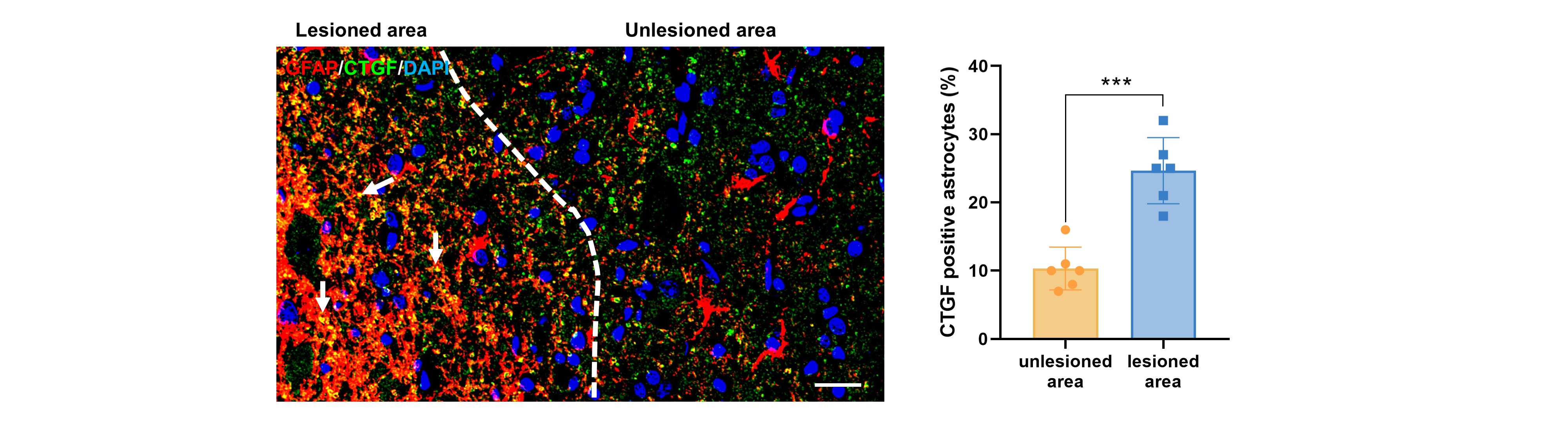


Figure S9. Immunofluorescence analysis of CTGF^+^ astrocytes in the spinal cord at 3 dpi. (a) Representative images showing CTGF (green) and GFAP (red) staining in lesioned and unlesioned areas. Cell nuclei stained with DAPI are shown in blue. (b) Quantification of CTGF^+^GFAP^+^ astrocytes (n = 6). Scale bar, 500 μm. Data are mean ± SEM. Statistical comparisons used unpaired two-tailed t-tests. ***p < 0.001.


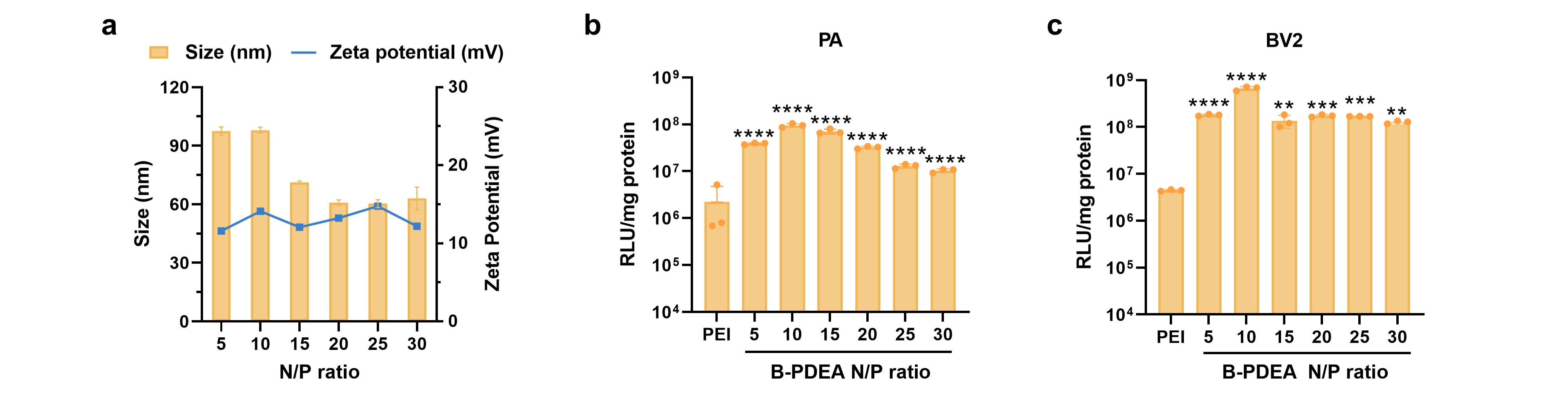


Figure S10. Physicochemical characterisation and transfection efficiency of B-PDEA/DNA polyplexes. (a) Volume-averaged size and zeta potential of B-PDEA/DNA polyplexes formed at varying N/P molar ratios (n = 3). (b, c) Luciferase expression in PAs (b) and BV2 microglial cells (c) following 48 h transfection with B-PDEA/pLuc polyplexes at a DNA dose of 2 μg/mL (n = 3). Data are mean ± SEM. Statistical comparisons used unpaired two-tailed t-tests, compared to PEI. **p < 0.01, ***p < 0.001, ****p< 0.0001.

**
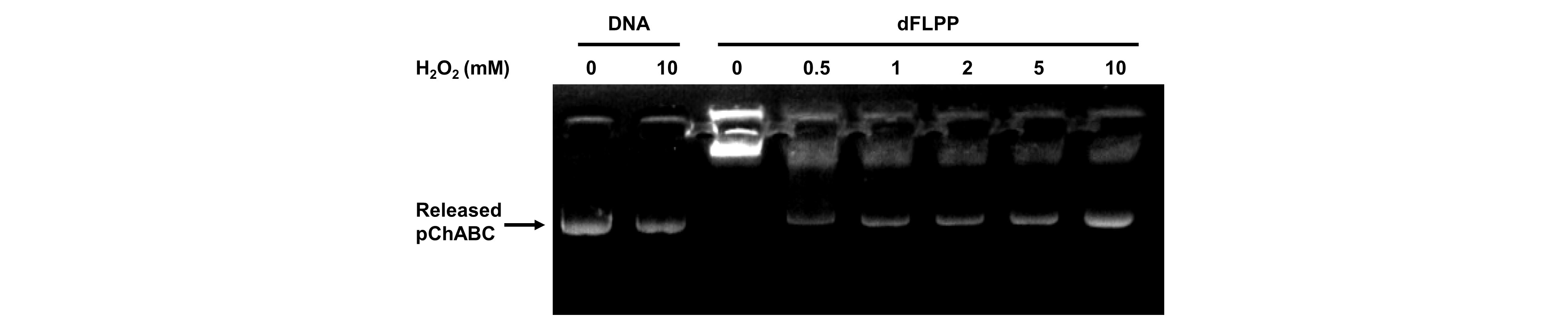
**

Figure S11. Gel retardation assay of dFLPP under varying H_2_O_2_ concentrations. dFLPP was incubated with 0, 0.5, 1, 2, 5, or 10 mM H_2_O­_2_ for 1 h at 37 ℃.


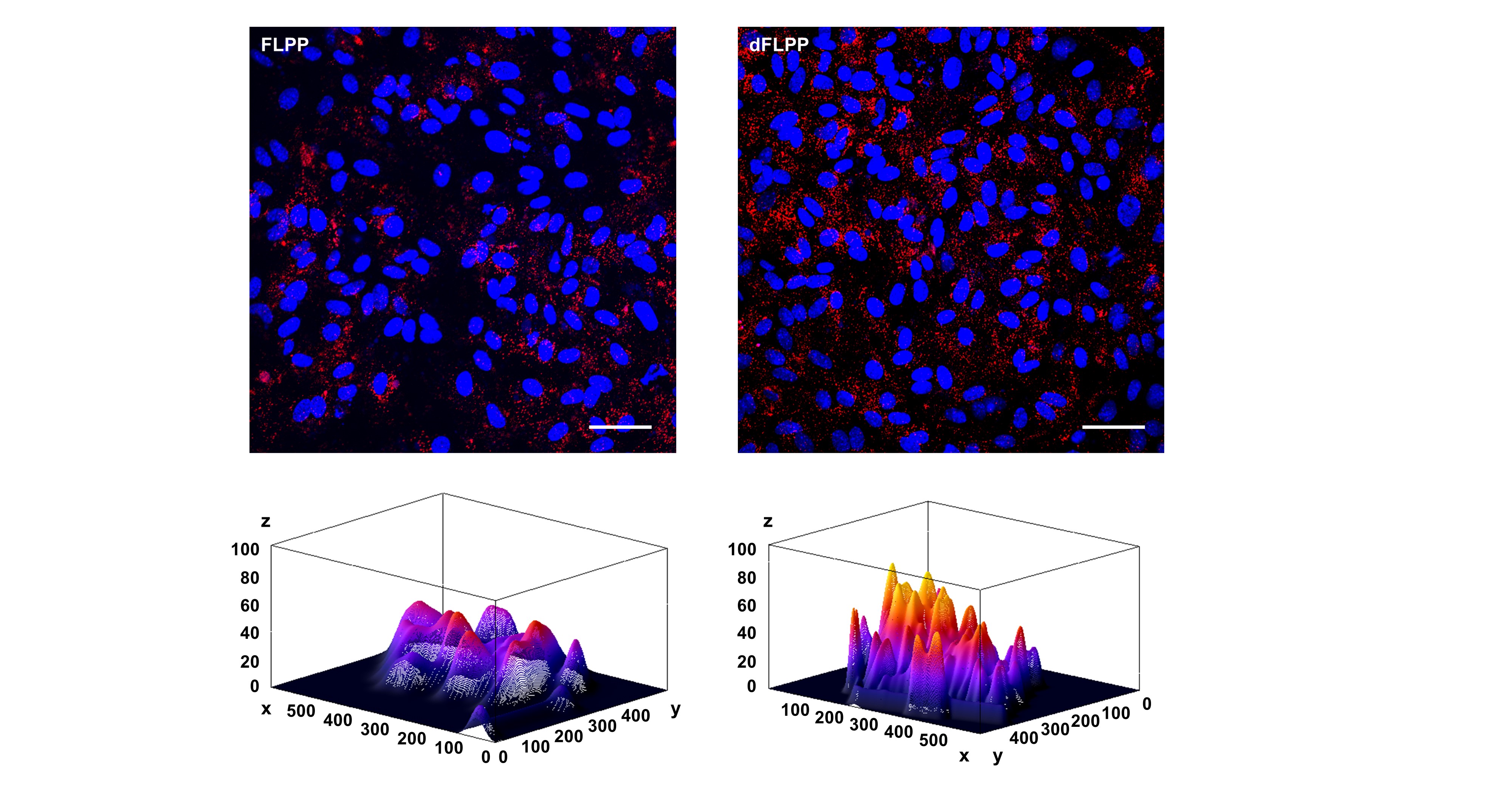


Figure S12. Confocal laser scanning microscopy (CLSM) images showing the cellular uptake of FLPP and dFLPP by RAs. PAs were stimulated with LPS (1 mg/mL) to induce an RA phenotype, followed by incubation with FLPP or dFLPP containing Cy5-labeled DNA (^Cy5^DNA, 1 μg/mL) for 1 h. ^Cy5^DNA is shown in red, and cell nuclei stained with Hoechst 33342 are shown in blue. Scale bars, 50 μm.


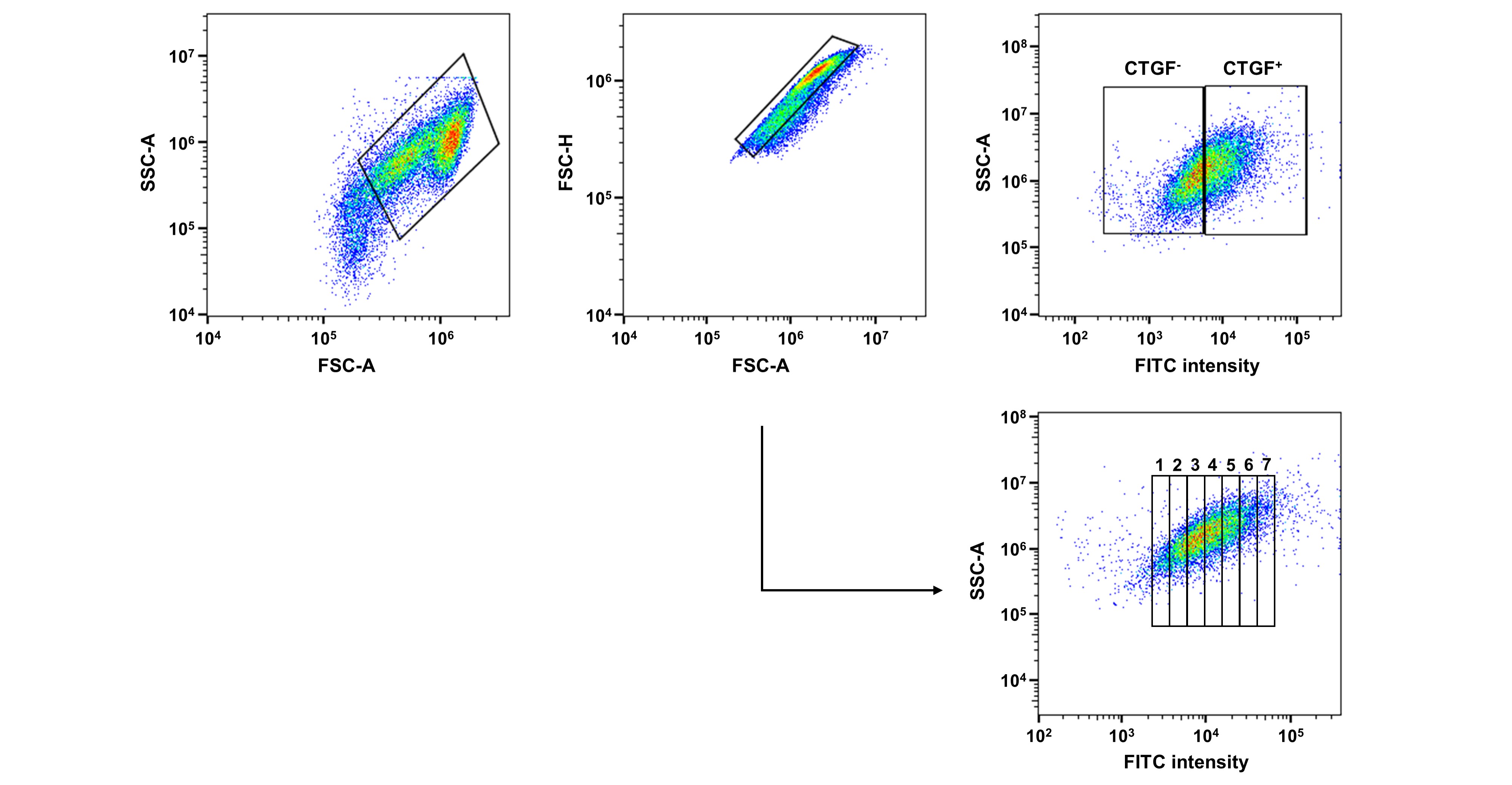


Figure S13. Flow cytometric gating strategy for analysing dFLPP uptake in CTGF^+^ astrocytes and correlation between CTGF expression and uptake efficiency. PAs were stimulated with LPS (1 mg/mL) for 18 h to induce a reactive phenotype, then mixed with untreated PAs at a 1:1 ratio to generate a heterogeneous astrocyte population. The mixed cells were subsequently incubated with dFLPP containing ^Cy5^DNA (1 μg/mL) for 1 h. CTGF expression was detected by immunofluorescent labeling using a primary anti-CTGF antibody and a FITC-conjugated secondary antibody. Astrocytes were grouped into seven subpopulations based on FITC fluorescence intensity, and the mean Cy5 signal was quantified within each group to evaluate the relationship between CTGF expression and dFLPP uptake.


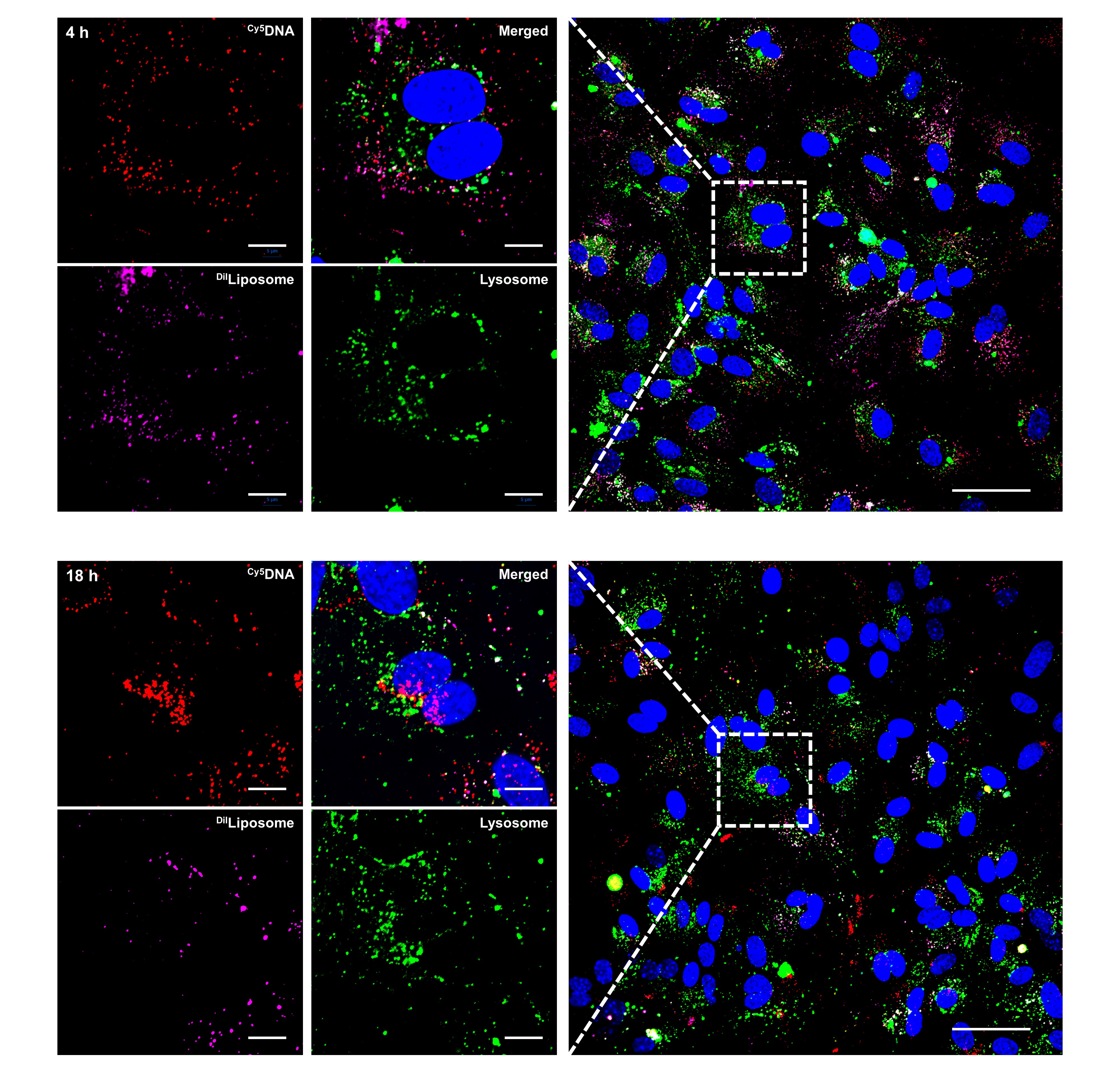


Figure S14. Intracellular distribution of dFLPP in RAs at 4 and 18 h, visualized by CLSM. RAs were incubated with dFLPP containing ^Cy5^DNA (1 μg/mL) and DiI-labeled lipids (0.05 μmol/μg DNA). ^Cy5^DNA is shown in red, DiI-labeled liposomes in purple, lysosomes in green (stained with LysoTracker), and nuclei in blue (Hoechst 33342). Scale bars, 10 μm (left); 50 μm (right).


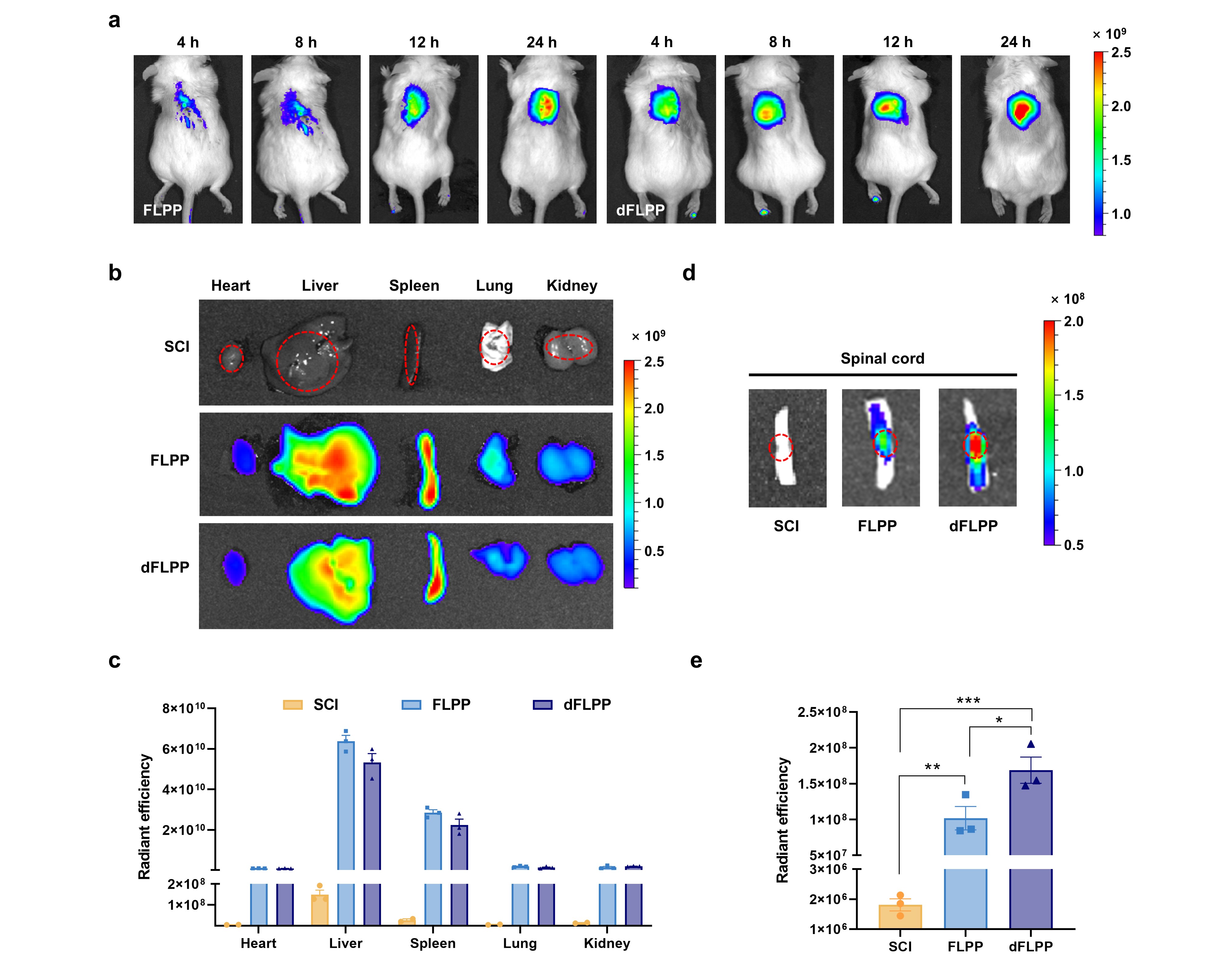


Figure S15. *In vivo* biodistribution of FLPP and dFLPP in spinal cord injury (SCI) mice. (a) *In vivo* fluorescence imaging of DiI-labeled FLPP and dFLPP at indicated time points following a single intravenous injection (pEGFP dose, 0.5 mg/kg; DiI, 0.05 μmol/μg DNA). (b, c) *Ex vivo* imaging (b) and fluorescence quantification (c) of major organs (heart, liver, spleen, lungs, kidneys) harvested 24 h post-injection (n = 3). (d, e) *Ex vivo* imaging (d) and corresponding fluorescence quantification (e) of spinal cords collected from SCI, DiI-labeled FLPP-, and dFLPP-treated groups (n = 3). Data are mean ± SEM. Statistical comparisons used one-way ANOVA with Tukey's post hoc test (≥ 3 groups). *p< 0.05, **p < 0.01, ***p < 0.001.

**
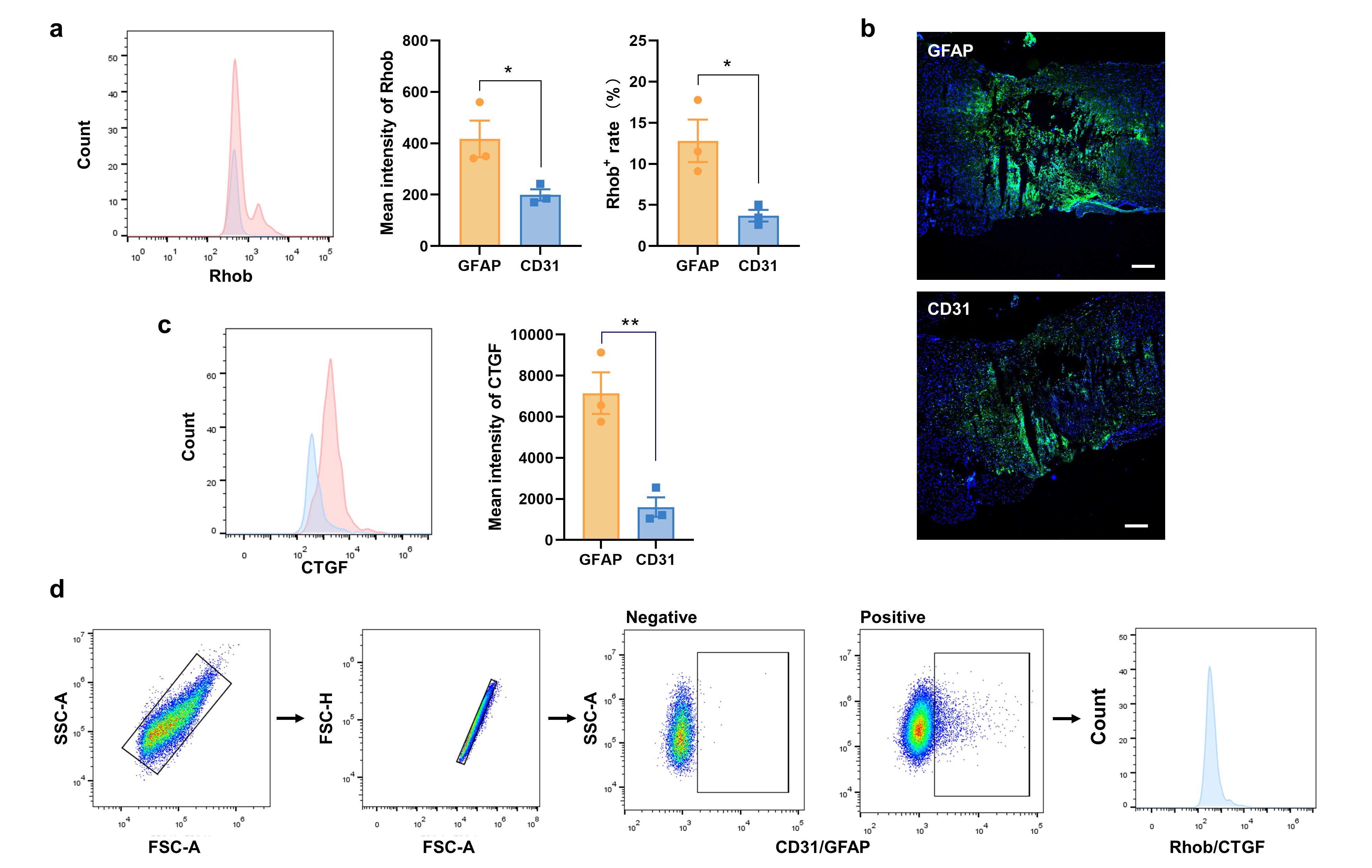
**

Figure S16. *In vivo* evaluation of cell-type specificity of dFLPP in the lesioned spinal cord. (a) Flow cytometric analysis of RhoB fluorescence intensity and positive cell rate within GFAP^+^ and CD31^+^ cell populations. SCI mice were *i.v.* injected with RhoB-labeled dFLPP (0.5 mg/kg) at 1 dpi, and spinal cord tissues were collected at 2 dpi, enzymatically dissociated, and immunostained for CD31 and GFAP (n = 3). (b) Spatial distribution of GFAP^+^ astrocytes and CD31^+^ endothelial cells in the lesion site at 2 dpi, assessed by immunofluorescence. Scale bar: 200 μm. (c) Comparison of CTGF expression levels in GFAP^+^ and CD31^+^ cells at 2 dpi (n=3). (d) The gating strategy used for flow cytometric analysis of RhoB-labeled dFLPP uptake and CTGF expression. Data are mean ± SEM. Statistical comparisons used unpaired two-tailed t-tests. *p< 0.05, **p < 0.01.

**
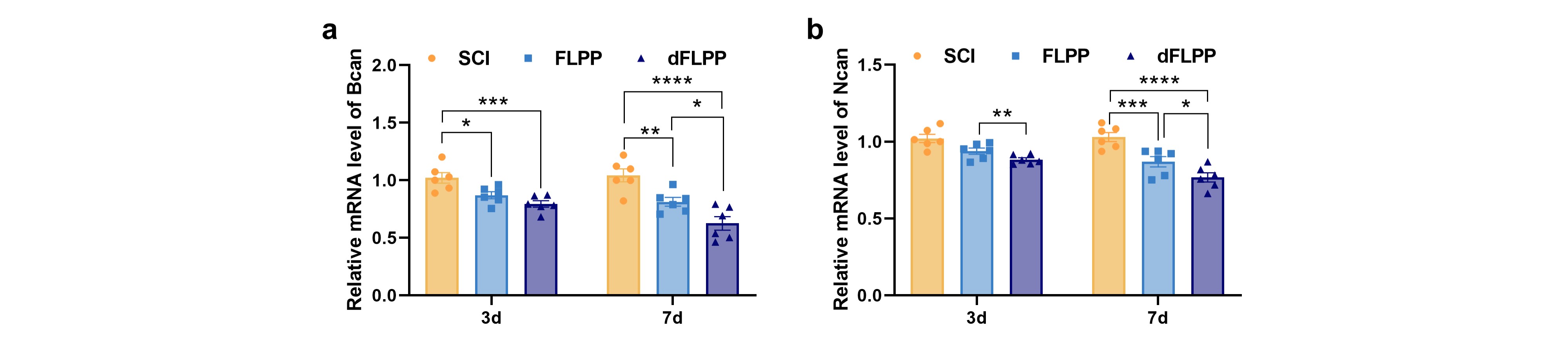
**

Figure S17. Quantitative RT-PCR analysis of mRNA expression levels of brevican (*Bcan*) (a) and neurocan (*Ncan*) (b) in spinal cord tissue at 3 and 7 dpi. SCI mice were intravenously administered FLPP or dFLPP at a pChABC dose of 0.5 mg/kg, starting at 1 dpi and repeated every two days for a total of one or three injections, respectively (n = 6). Data are mean ± SEM. Statistical comparisons used one-way ANOVA with Tukey's post hoc test (≥ 3 groups). *p < 0.05, **p < 0.01, ***p < 0.001, ****p < 0.0001.


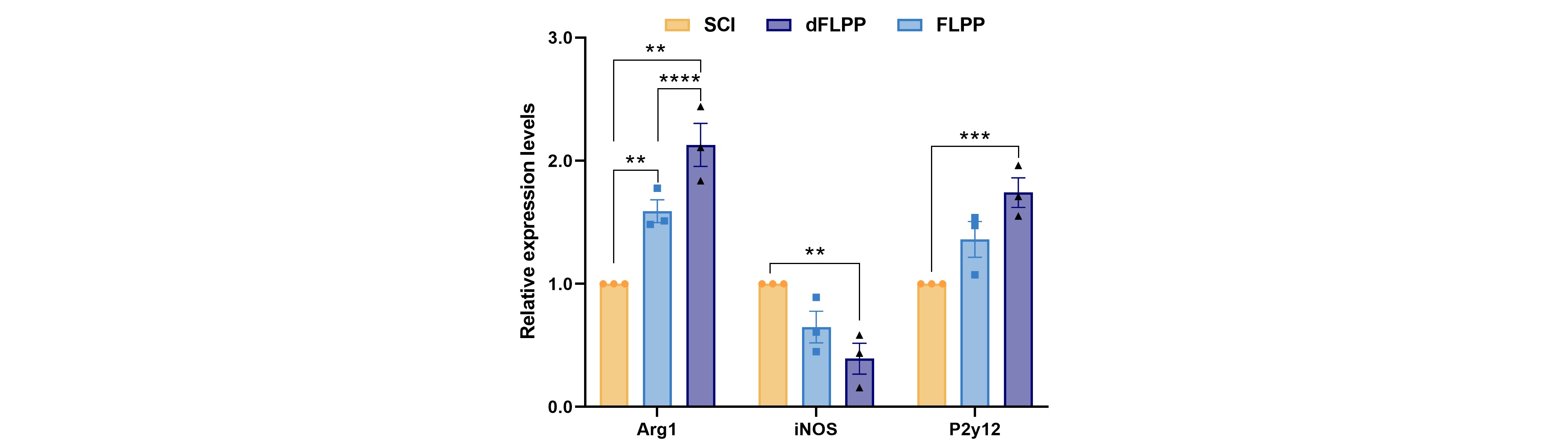


Figure S18. Quantification of Arg1, iNOS, and P2y12 expression in Figure 6h using ImageJ (n = 3). Data are mean ± SEM. Statistical comparisons used one-way ANOVA with Tukey's post hoc test (≥ 3 groups). *p < 0.05, **p < 0.01, ***p < 0.001, ****p < 0.0001.


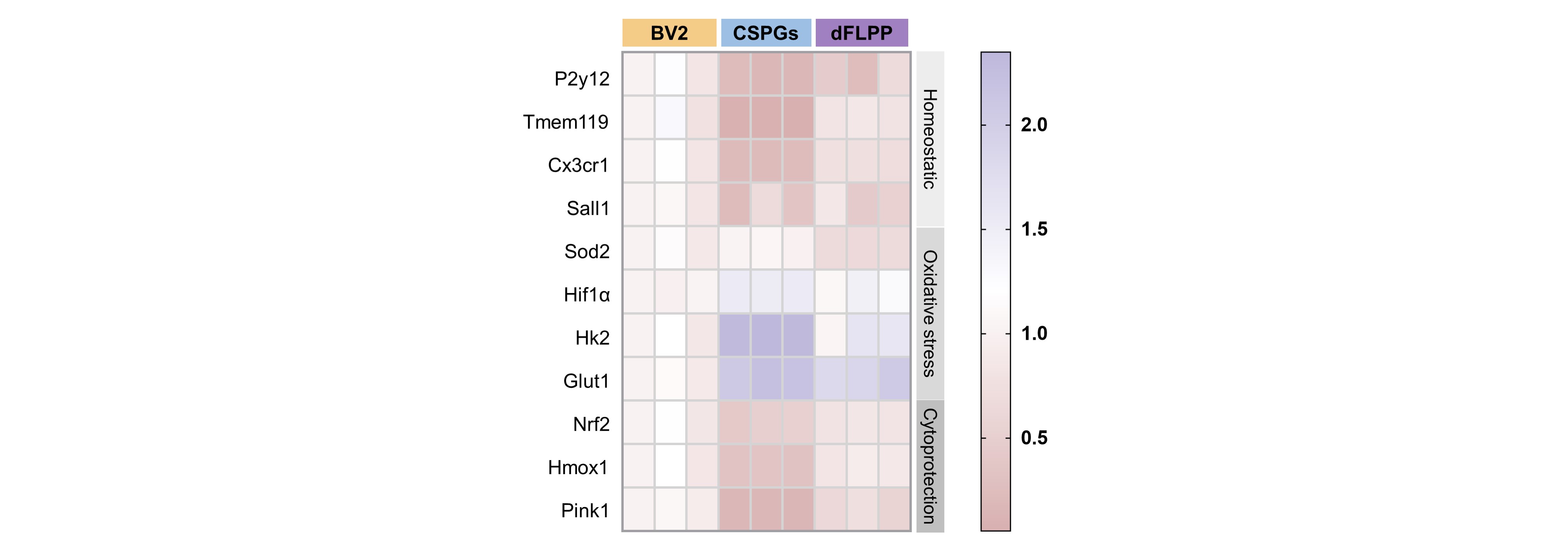


Figure S19. Transcriptomic profiling of microglial phenotypic markers following CSPG stimulation and dFLPP treatment. The expression levels of genes associated with homeostatic microglial identity, oxidative stress and metabolic reprogramming, and cytoprotective responses were assessed in primary microglia by transcriptomic analysis. Cells were left untreated, stimulated with CSPGs (3.34 μg/mL), or co-treated with CSPGs and dFLPP (2 μg/mL pChABC) for 72 h (n = 3).


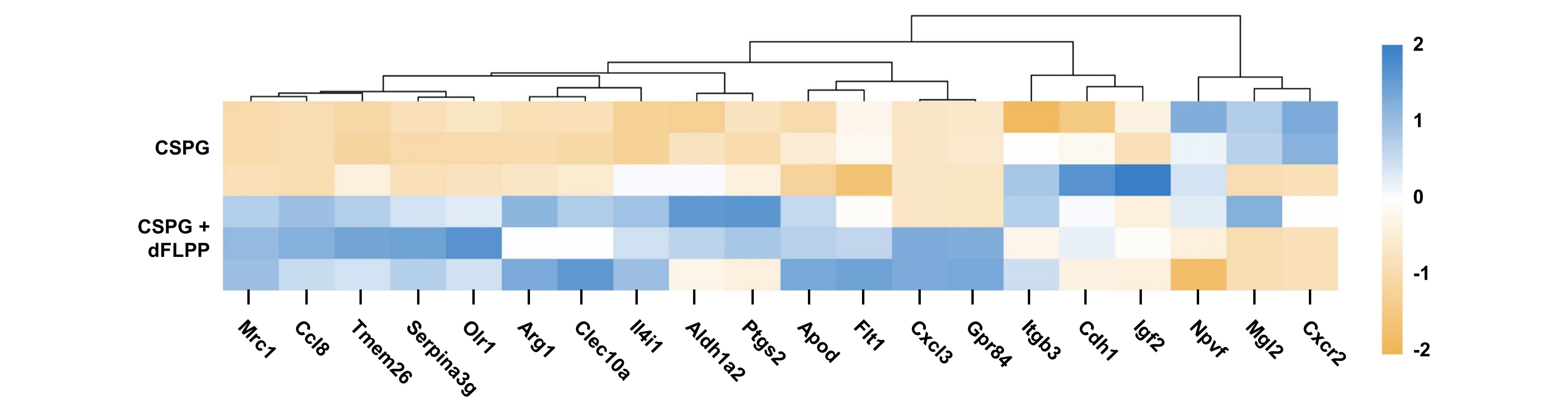


Figure S20. Transcriptomic analysis of anti-inflammatory gene expression in PMs following CSPG stimulation and dFLPP treatment. PMs were treated with CSPGs (3.34 µg/mL) or co-treated with CSPGs and dFLPP (2 μg/mL pChABC) for 72 h. Gene expression profiles were visualized as a heatmap, with hierarchical clustering performed using the Ward.D2 method (n = 3).


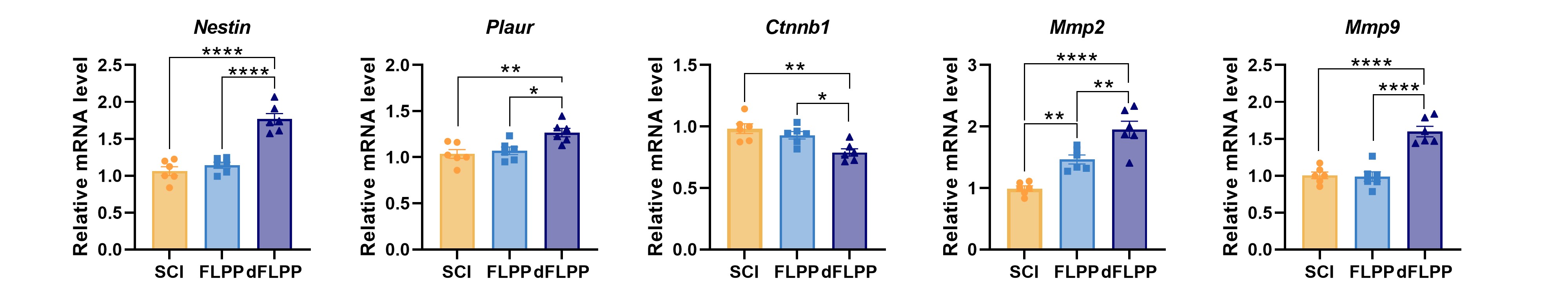


Figure S21. Quantitative RT-PCR analysis of mRNA expression levels of RA-associated genes in spinal cords at 28 dpi from SCI, FLPP- or dFLPP-treated mice (n = 6). Data are mean ± SEM. Statistical comparisons used one-way ANOVA with Tukey's post hoc test (≥ 3 groups). *p < 0.05, **p < 0.01, ***p < 0.001, ****p < 0.0001.


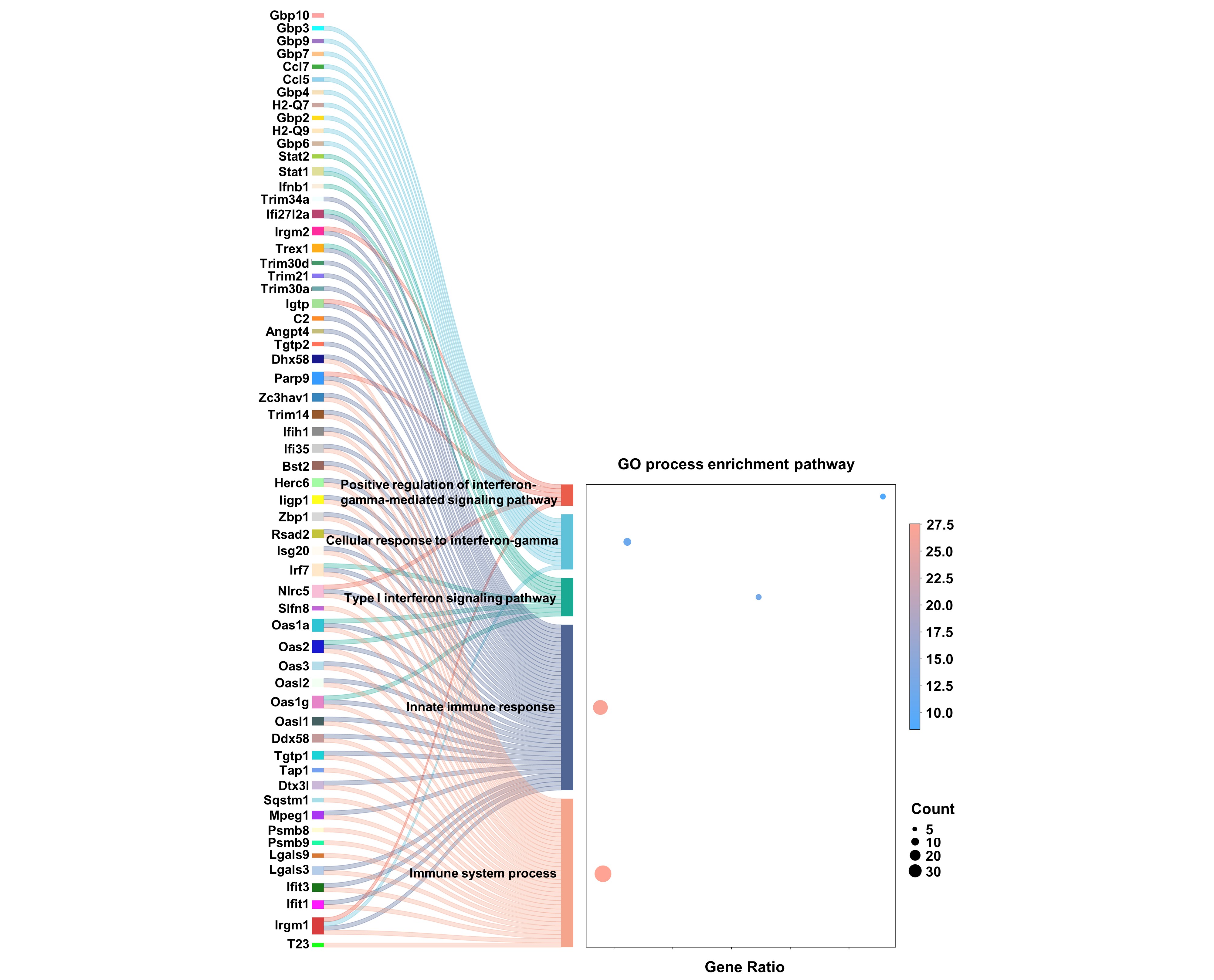


Figure S22. Sankey diagram of enriched GO biological processes in dFLPP-treated PAs under CSPG stimulation. GO pathway enrichment analysis was performed on DEGs upregulated in PAs following dFLPP treatment under CSPG exposure (3.34 µg/mL), based on transcriptomic data.


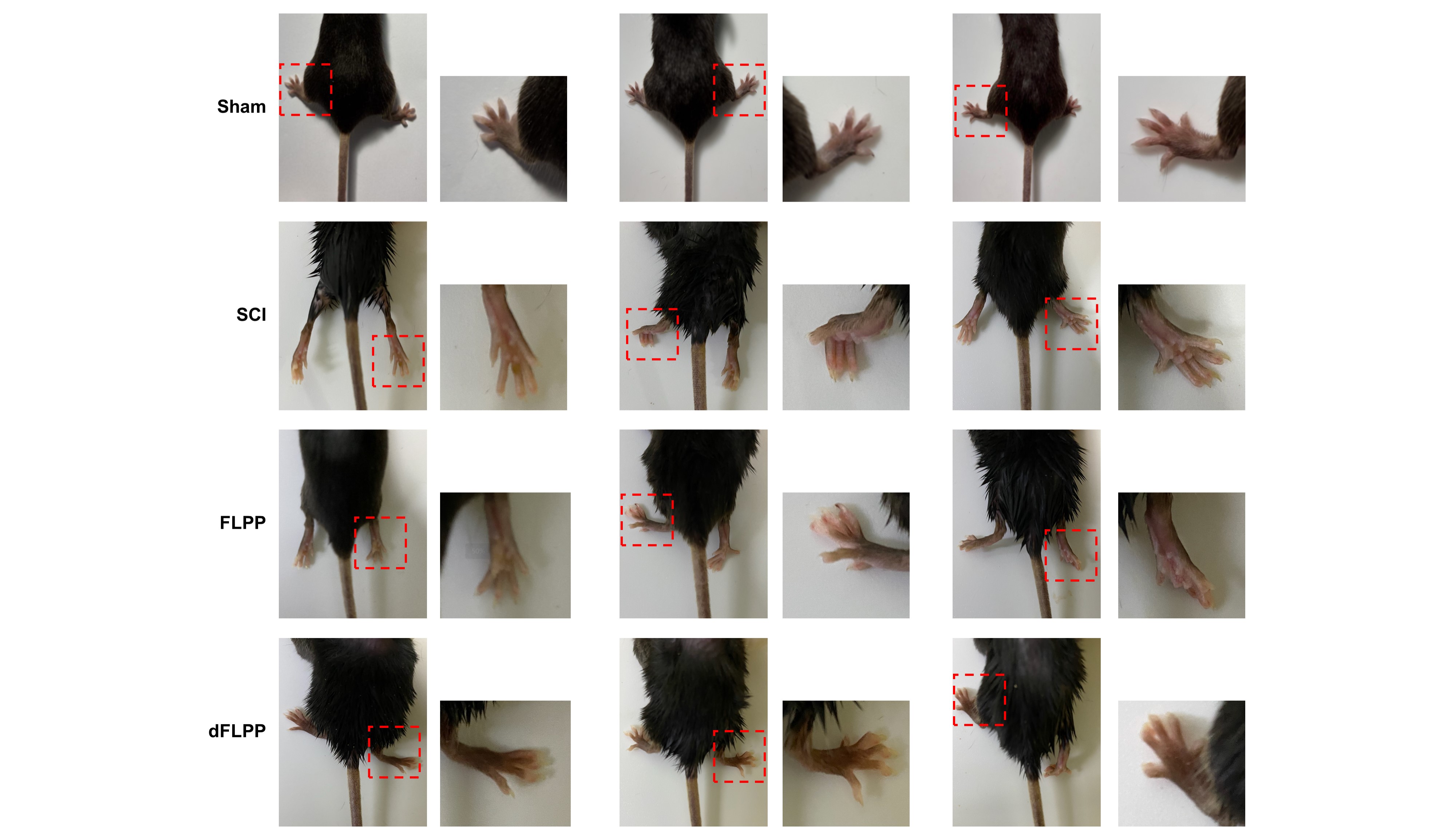


Figure S23. Representative images of hind paws from Sham, SCI, FLPP-, or dFLPP-treated mice at 28 dpi.


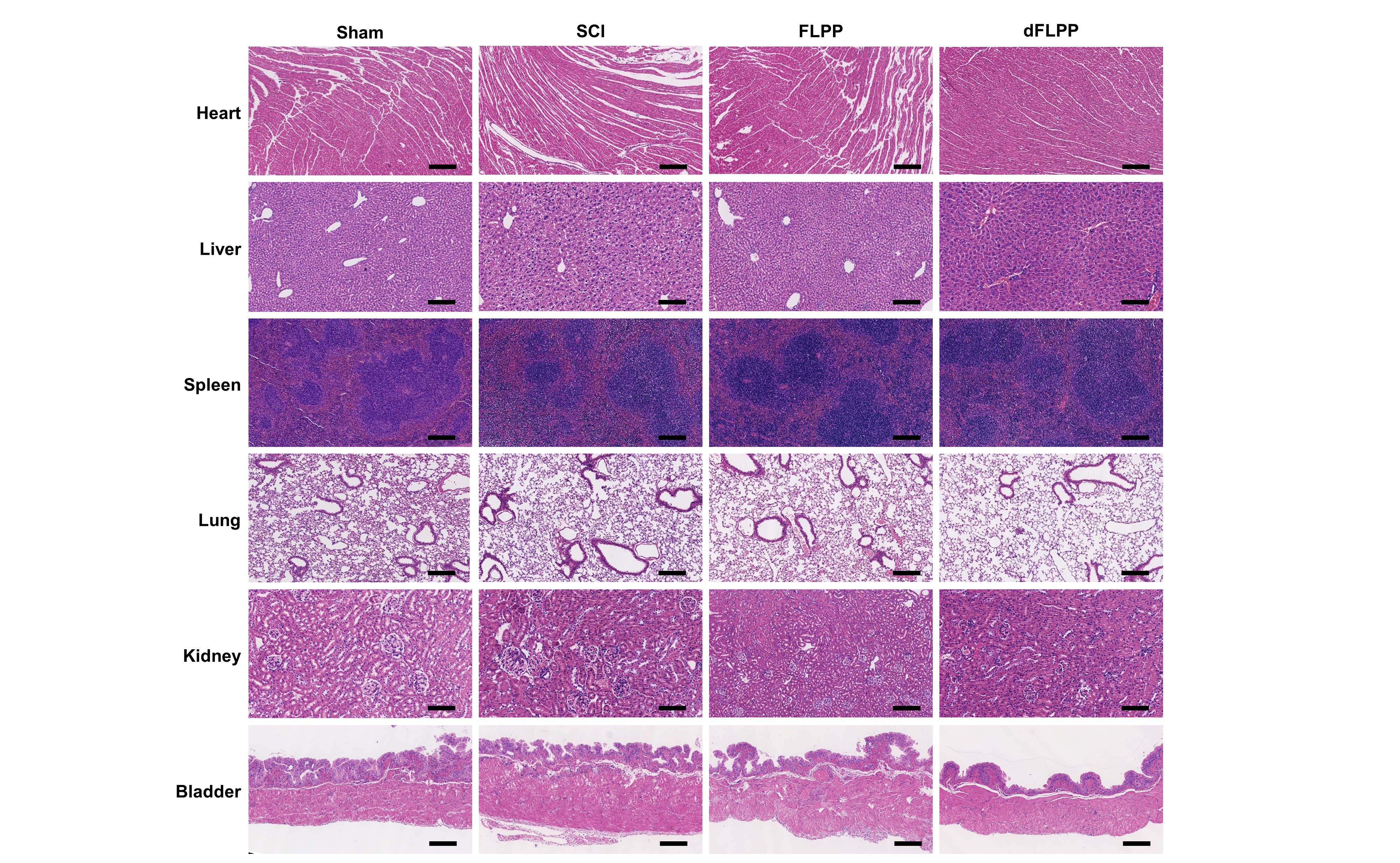


Figure S24. Representative haematoxylin and eosin (H&E) staining images of the heart, liver, spleen, lung, kidney, and bladder collected from Sham, SCI, FLPP-, or dFLPP-treated mice at 28 dpi. Scale bars, 200 μm.


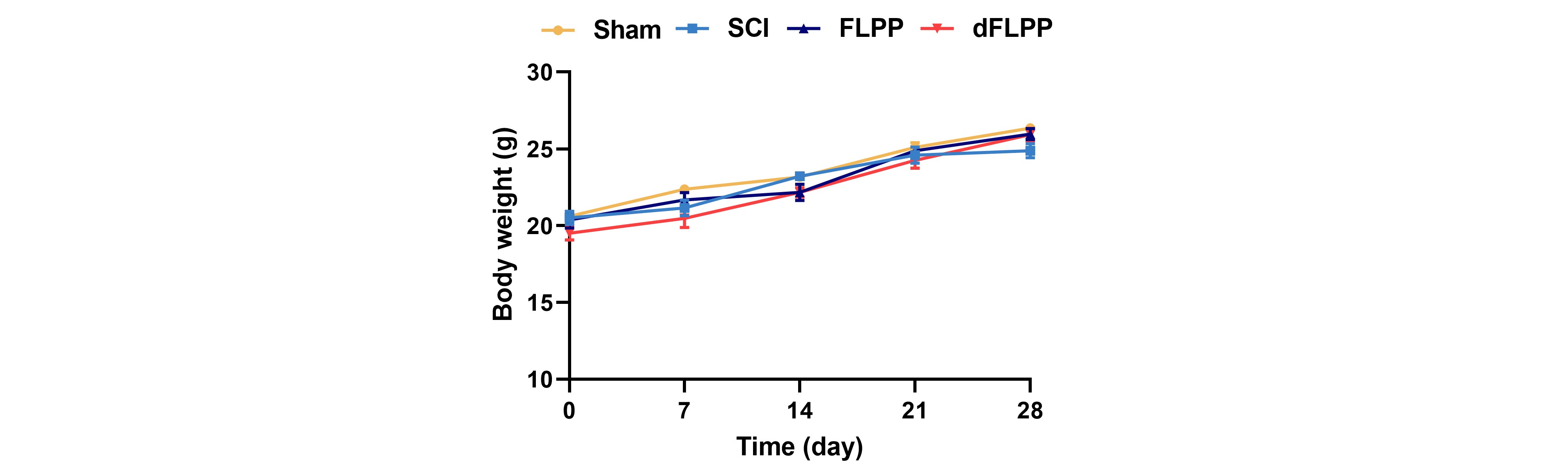


Figure S25. Body weight changes of mice in Sham, SCI, FLPP-, and dFLPP-treated groups throughout the experimental period (n = 6).


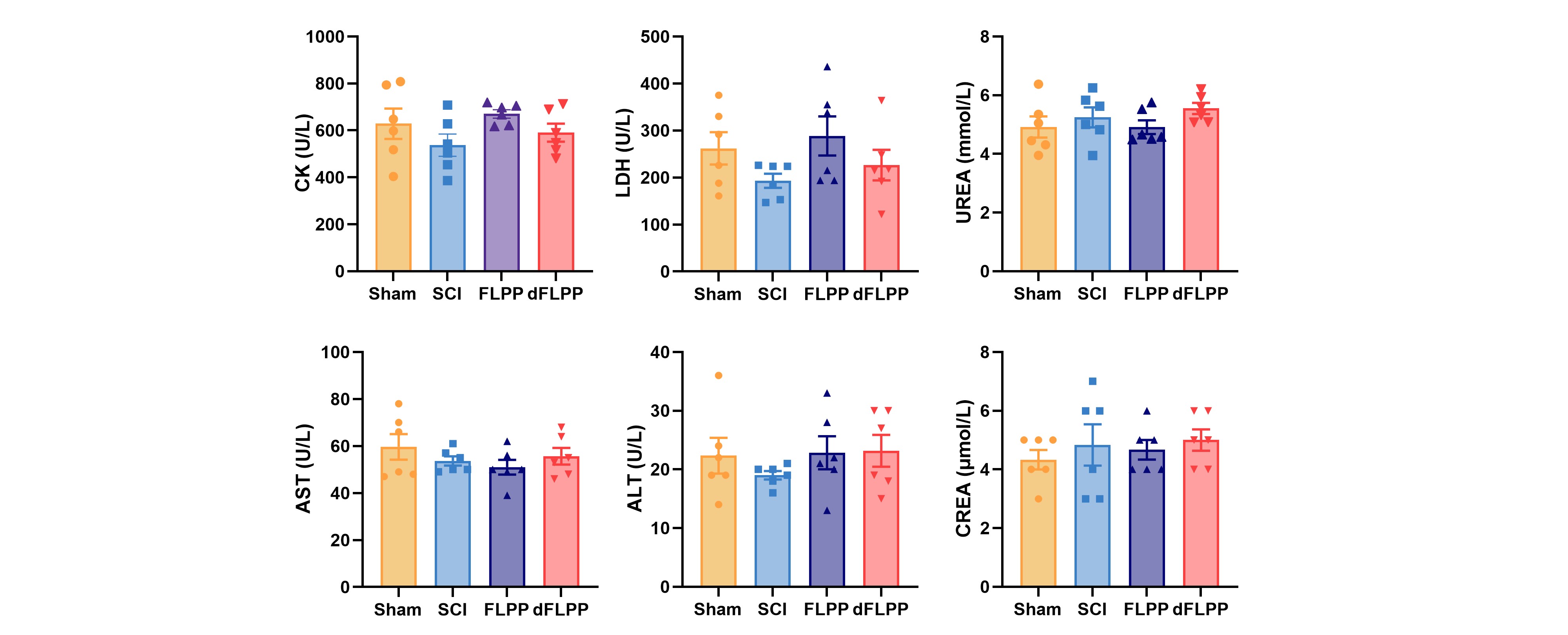


Figure S26. Evaluation of systemic biosafety following dFLPP treatment at 28 dpi. SCI mice received *i.v.* FLPP or dFLPP starting at 1 dpi (pChABC dose, 0.5 mg/kg), administered every other day for a total of five doses. At 28 dpi, blood samples were collected, and serum biomarkers of cardiac (CK, LDH), hepatic (AST, ALT), and renal (CREA, UREA) function were quantified (n = 6).


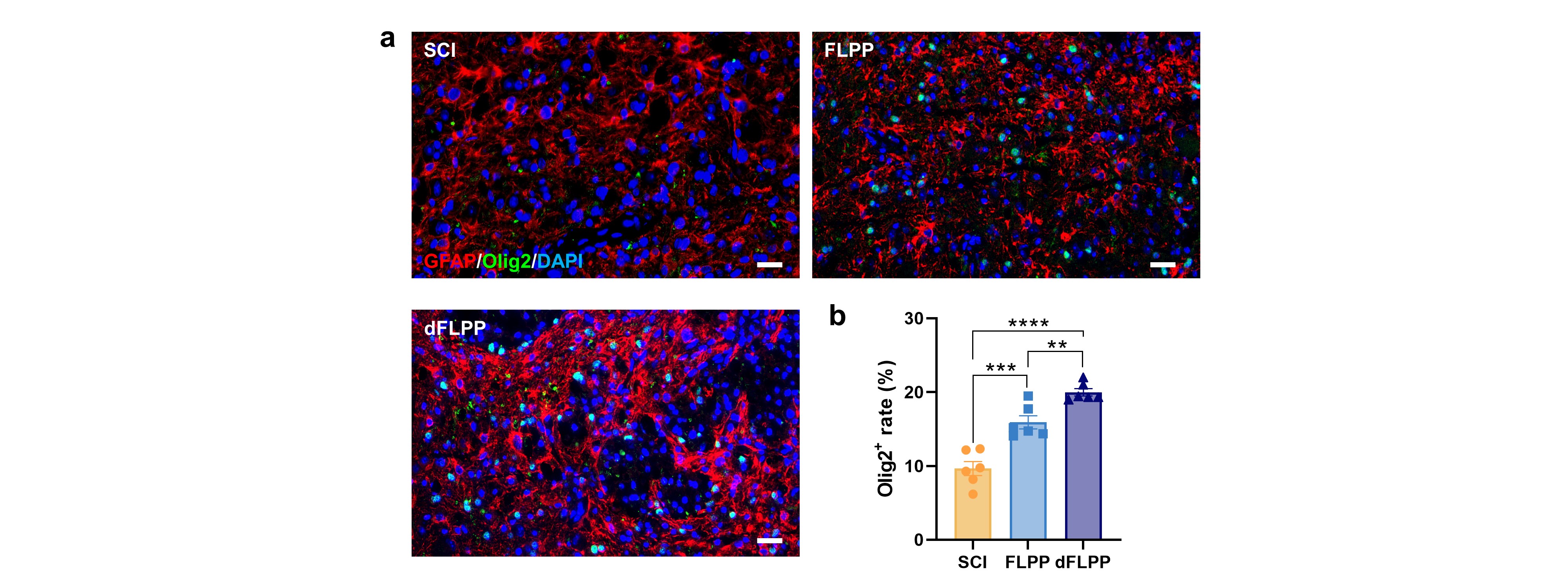


Figure S27. Representative immunofluorescent images of oligodendrocyte transcription factor 2 (Olig2, green), GFAP (red), and nuclei (blue) in spinal cord sections (a) and quantification of Olig2⁺ cells in the lesioned area (b) (n = 6). Scale bars, 20 μm. Data are mean ± SEM. Statistical comparisons used one-way ANOVA with Tukey's post hoc test (≥ 3 groups). **p < 0.01, ***p < 0.001, ****p < 0.0001.

Table S1. qRT-PCR primers

| **Genes** | **Forward 5'-3'** | **Reverse 5'- 3'** |
| --- | --- | --- |
| *Chst11* | GATGAACAGAATTTGCCGGATGG | GTTCCTCCGCATGACTGGG |
| *Xylt1* | TCCACAACCAAGTCCACTGAT | GCGAAGTTGCTGTTATCCACATT |
| *Sox9* | CGGAACAGACTCACATCTCTCC | GCTTGCACGTCGGTTTTGG |
| *Acan* | CCTGCTACTTCATCGACCCC | AGATGCTGTTGACTCGAACCT |
| *Bcan* | TGCGCGTCAAGGTAAACGAA | GACCCCGGAATCATTGGGC |
| *Ncan* | TGCAACCACGGCTAAGCTC | GGGGATAAGCAGGCAATGAC |
| *Pcan* | GCCCCGAGGAGAATGAATTTG | GAAACTCACGCAGGTCAATGC |
| *Vcan* | TTTTACCCGAGTTACCAGACTCA | GGAGTAGTTGTTACATCCGTTGC |
| *Cdh2* | AGCGCAGTCTTACCGAAGG | TCGCTGCTTTCATACTGAACTTT |
| *ChABC* | AGAGCCGTAGGCGTCTCTCT | AGCGTTGAGGGTCATCTCTC |
| *Arg1* | CTCCAAGCCAAAGTCCTTAGAG | AGGAGCTGTCATTAGGGACATC |
| *iNOS* | GTTCTCAGCCCAACAATACAAGA | GTGGACGGGTCGATGTCAC |
| *P2y12* | ATGGATATGCCTGGTGTCAACA | AGCAATGGGAAGAGAACCTGG |
| *Axin* | GAGAAGTTGGATTGCTGTCCACG | ACCATTTCTGCATGTGTCGATGG |
| *Mmp9* | CTGGACAGCCAGACACTAAAG | CTCGCGGCAAGTCTTCAGAG |
| *Mmp2* | CAAGTTCCCCGGCGATGTC | TTCTGGTCAAGGTCACCTGTC |
| *Nestin* | CCCTGAAGTCGAGGAGCTG | CTGCTGCACCTCTAAGCGA |
| *Ctnnb* | ATGGAGCCGGACAGAAAAGC | CTTGCCACTCAGGGAAGGA |
| *CD86* | AGACATGTGTAACCTGCACCAT | ACCGACTTTTTCCGGTCCTG |
| *CD206* | GTGGAGTGATGGAACCCCAG | CTGTCCGCCCAGTATCCATC |
| *Col1a1* | GCAAGAGGCGAGAGAGGTTT | GACCACGGGCACCATCTTTA |
| *Col2a1* | CTGGAACAAATGGGCTCACTG | CAGGCTCACCAACAAGTCCTC |
| *Ifn-γ* | ATGAACGCTACACACTGCATC | CCATCCTTTTGCCAGTTCCTC |
| *Tnf-α* | CCCTCACACTCAGATCATCTTCT | GCTACGACGTGGGCTACAG |
| *Tgf-β* | CTCCCGTGGCTTCTAGTGC | GCCTTAGTTTGGACAGGATCTG |
| *Il-1β* | GCAACTGTTCCTGAACTCAACT | ATCTTTTGGGGTCCGTCAACT |
| *Tmem119* | CCTACTCTGTGTCACTCCCG | CACGTACTGCCGGAAGAAATC |
| *Cx3cr1* | GAGTATGACGATTCTGCTGAGG | CAGACCGAACGTGAAGACGAG |
| *Sall1* | CTCAACATTTCCAATCCGACCC | GGCATCCTTGCTCTTAGTGGG |
| *Sod2* | CAGACCTGCCTTACGACTATGG | CTCGGTGGCGTTGAGATTGTT |
| *Hif1α* | ACCTTCATCGGAAACTCCAAAG | ACTGTTAGGCTCAGGTGAACT |
| *Hk2* | TGATCGCCTGCTTATTCACGG | AACCGCCTAGAAATCTCCAGA |
| *Nrf2* | TCTTGGAGTAAGTCGAGAAGTGT | GTTGAAACTGAGCGAAAAAGGC |
| *Hmox1* | AAGCCGAGAATGCTGAGTTCA | GCCGTGTAGATATGGTACAAGGA |
| *Cyp1a1* | GACCCTTACAAGTATTTGGTCGT | GGTATCCAGAGCCAGTAACCT |
| *Cyp1b1* | CACCAGCCTTAGTGCAGACAG | GAGGACCACGGTTTCCGTTG |
| *Cyp2d22* | CAGTGGTTGTACTAAATGGGCT | GCTAGGACTATACCTTGAGAGCG |
| *Gsta1* | AAGCCCGTGCTTCACTACTTC | GGGCACTTGGTCAAACATCAAA |
| *Gsta3* | AAGAATGGAGCCTATCCGGTG | CCATCACTTCGTAACCTTGCC |
| *Ugt1a1* | GCTTCTTCCGTACCTTCTGTTG | GCTGCTGAATAACTCCAAGCAT |
| *Glut1* | CAGTTCGGCTATAACACTGGTG | GCCCCCGACAGAGAAGATG |
| *Gapdh* | AGGTCGGTGTGAACGGATTTG | TGTAGACCATGTAGTTGAGGTCA |

Table S2. Antibody information and concentrations

| **Antibody** | **Source (cat#)** | **Usage, Dilution** |
| --- | --- | --- |
| Anti-GFAP antibody | Abcam (ab4674, ab279290) | IF, 1:200; Flow cyt, 1:500 |
| Anti-NeuN antibody | Abcam (ab177487) | IF, 1:100 |
| Anti-GAP-43 antibody | Abcam (ab75810) | IF, 1:200 |
| Anti-NF-200 antibody | Abcam (ab207176) | IF, 1:100 |
| Anti-Nestin antibody | Santa Cruz (sc23927) | IF, 1:100 |
| Anti-ChAT antibody | Abcam (ab181023) | IF, 1:100 |
| Anti-5-HT antibody | Abcam (ab254358) | IF, 1:50 |
| Anti-Olig2 antibody | Abcam (ab109186) | IF, 2.5μg/mL |
| Anti-Iba1 antibody | Abcam (ab283346) | IF, 1:100 |
| Anti-iNOS antibody | Abcam (ab178945) | IF, 1:500; WB, 1:1000 |
| Anti-Arg1 antibody | Abcam (ab96183) | IF, 1:200; WB, 1:1000 |
| Anti-CS56 antibody | Abcam (ab11570) | IF, 1:200 |
| Anti-P2y12 antibody | Invitrogen (4H5L19) | IF, 1:200; WB, 1:1000 |
| Anti-Fibronectin antibody | Abcam (ab268020) | IF, 1:50 |
| Anti-CTGF antibody | Abcam (ab318148) | IF, 1:500; Flow cyt, 1:500 |
| Anti-SOX9 antibody | Abcam (ab185966) | IF, 2.5μg/Ml; Flow cyt, 1:500 |
| Anti-NG2 antibody | Abcam (ab275024) | WB, 1:1000 |
| Anti-ChABC antibody | Abcam (ab256485) | WB, 0.092 µg/mL |
| Anti-MMP2 antibody | Abcam (ab92536) | Flow cyt, 1:200 |
| Anti-CD31 antibody | Abcam (ab7388) | IF, 1:100; Flow cyt,1:500 |
| Goat Anti-Chicken IgY H&L (Alexa Fluor® 647) | Abcam (ab150176) | IF, 1:500 |
| Goat Anti-Mouse IgG H&L (Alexa Fluor® 488) | Abcam (ab150113) | IF, 1:500; Flow cyt, 1:2000 |
| Donkey Anti-Rabbit IgG H&L (Alexa Fluor® 647) | Abcam (ab150075) | IF, 1:500; Flow cyt, 1:2000 |
| Donkey Anti-Rabbit IgG H&L (Alexa Fluor® 488) | Abcam (ab150073) | IF, 1:500 |
| HRP Anti-Rabbit IgG antibody | Abcam (ab288151) | WB, 1:5000 |
| Goat Anti-Mouse IgG H&L (HRP) | Abcam (ab205719) | WB, 1:5000 |

References

1. X. Liu, J. Xiang, D. Zhu, L. Jiang, Z. Zhou, J. Tang, X. Liu, Y. Huang, Y. Shen, *Adv. Mater.* 2015, 28, 1743–1752.
2. X. Liu, Y. Zheng, Q. Wang, L. Zhao, Z. Zhang, H. Wang, Y. Yang, N. Song, J. Xiang, Y. Shen, S. Fan, J. Control. Release 2023, 364, 601–617.
3. V. Veneruso, E. Petillo, F. Pizzetti, A. Orro, D. Comolli, M. De Paola, A. Verrillo, A. Baggiolini, S. Votano, F. Castiglione, M. Sponchioni, G. Forloni, F. Rossi, P. Veglianese, Adv. Mater. 2024, 36, 2307747.
4. L. Yuan, S. Liu, X. Bai, Y. Gao, G. Liu, X. Wang, D. Liu, T. Li, A. Hao, Z. Wang, J. Neuroinflammation 2016, 13, 77.
5. Z. Ru, M. Xu, G. Zhu, Y. Tu, Y. Jiang, H. Du, Food Sci. Nutr. 2021, 9, 6162–6175.
6. Y. Zheng, Z. Zhang, Z. Fu, A. Fan, N. Song, Q. Wang, S. Fan, J. Xu, J. Xiang, X. Liu, ACS Nano 2024, 18, 26153–26167.
